# Synthesis, Antibacterial Activities, Mode of Action and Acute Toxicity Studies of New Oxazolidinone-Fluoroquinolone Hybrids

**DOI:** 10.3390/molecules24081641

**Published:** 2019-04-25

**Authors:** Lili Liu, Liping Shao, Jing Li, Haifeng Cui, Bing Li, Xuzheng Zhou, Pengyue Lv, Jiyu Zhang

**Affiliations:** 1 Key Laboratory of Veterinary Pharmaceutical Development, Ministry of Agriculture, Lanzhou 730050, China; liull0728@163.com (L.L.); shaolp1992@163.com (L.S.); binglilb@163.com (B.L.); zhouxuzh@126.com (X.Z.); 2 Key Laboratory of New Animal Drug project of Gansu Province, Lanzhou 730050, China; 3 Lanzhou Institute of Husbandry and Pharmaceutical Sciences, Chinese Academy of Agricultural Sciences (CAAS), Lanzhou 730050, China; 4 R & D Center, Beijing Orbiepharm Co., Ltd., Beijing 100085, China; lijing1958@126.com (J.L.); cuihf1981@163.com (H.C.); lvpenyue@163.com (P.L.)

**Keywords:** oxazolidinone-fluoroquinolone hybrids, synthesis, antibacterial activity, mode of action, acute toxicity

## Abstract

To combat bacterial resistance, a series of new oxazolidinone-fluoroquinolone hybrids have been synthesized and characterized. All synthetic hybrids were preliminarily evaluated for their in vitro antibacterial activities against 6 standard strains and 3 clinical isolates. The majority of hybrids displayed excellent activities against Gram-positive bacteria, but limited activities against Gram-negative bacteria. Hybrids OBP-4 and OBP-5 were found to be the most promising compounds. Further, in vitro antibacterial activities, mode of action and acute toxicity in mice of hybrids OBP-4 and OBP-5 were investigated. Hybrids OBP-4 and OBP-5 exhibited potent activities against Gram-positive bacteria, including drug-resistant strains. Correspondingly, studies on the mode of action of hybrids OBP-4 and OBP-5 indicated a strong inhibitory activity on protein synthesis by binding the active site of 50S subunit, but a weak inhibitory action on DNA synthesis. In addition, LD_50_ values of hybrids OBP-4 and OBP-5 in the acute oral toxicity were larger than 2000 mg/kg, suggesting a good safety profile.

## 1. Introduction

Although the discovery of antibiotics has had a profound positive impact on the improvements of human health and life expectancy, the emergence of antibiotic resistance has severely limited their clinical efficacy and applications [1,2]. Drug-resistant bacteria, especially multi-drug resistant (MDR) bacteria, representing the most common causative agents of nosocomial infections [3,4], are difficult to deal with using currently existing antibiotics. Worse still, the continuous evolution and spread of antibiotic-resistant bacteria have caused enormous financial burden worldwide in terms of increased morbidity, mortality and use of medical resources [5,6,7]. It is estimated that antibiotic-resistant infections could claim 10 million deaths per year and up to 100 trillion US dollars by 2050 if nothing is done [8]. Nowadays, not only is antimicrobial resistance a major public concern, but more worryingly, there is an urgent need for novel antibacterials with improved potency and a low propensity for resistance development.

In recent years, hybrid antibacterials, linking two different pharmacophores by a non-cleavable spacer, were introduced as a promising strategy to fight drug-resistant bacteria and delay resistance development [9,10]. Such a strategy has been applied to the design and synthesis of various hybrid antibacterials [11,12,13,14]. Fluoroquinolone, with its superior dual-target activity, chemical stability and distinct structure-activity relationship (SAR), is the most popular partner in the design of hybrid antibacterials [15]. Oxazolidinone exerted antibacterial activity by inhibiting protein synthesis [16], and has a dissimilar mechanism of action to fluoroquinolone. The covalent combination of oxazolidinone and fluoroquinolone may be a better choice for fighting the prevalent resistance to these two classes of compounds [17,18]. Furthermore, their SAR investigations have shown that oxazolidinone and fluoroquinolone have a good tolerance for structural modification and a common heterocyclic amine was introduced at the 4- and 7- positions of the respective phenyl ring [19], which provide an advantage for their combination. Meanwhile, previous studies have demonstrated that the oxazolidinone-quinolone hybrids with a 4-piperidinemethanol linker exhibited improved antibacterial activities [20] and a low propensity for spontaneous resistance development [21,22]. 

Inspired by these previous studies, we synthesized a series of new oxazolidinone-fluoroquinolone hybrids with the modifications of the spacer or fluoroquinolone pharmacophore. Here, we report the in vitro antibacterial activities of these hybrids, and the mode of action and acute toxicity of hybrids OBP-4 and OBP-5.

## 2. Results and Discussion

### 2.1. Chemistry

**Scheme 1:**
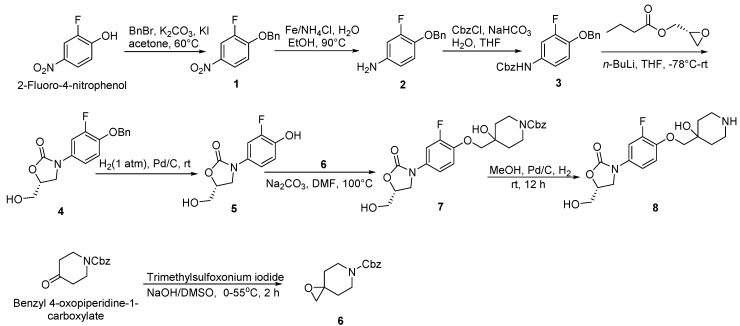
Synthesis of compounds **6** and **8**.

**Scheme 2:**
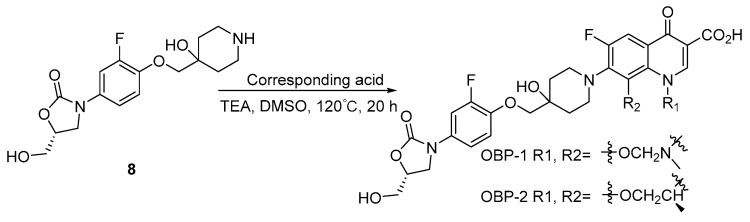
Synthesis of hybrids OBP-1 and OBP-2.

**Scheme 3:**
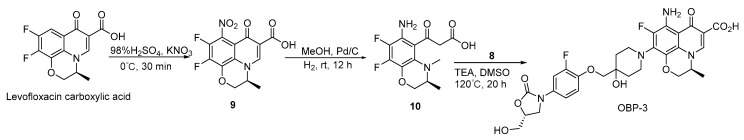
Synthesis of hybrid OBP-3.

**Scheme 4:**
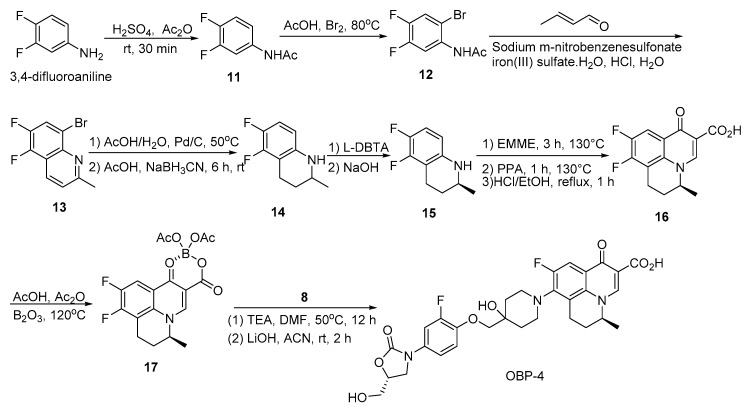
Synthesis of hybrid OBP-4.

**Scheme 5:**
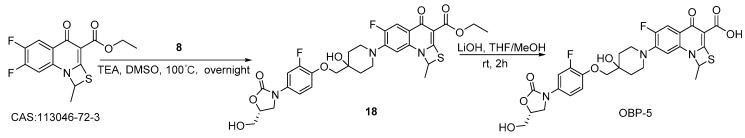
Synthesis of hybrid OBP-5.

**Scheme 6:**
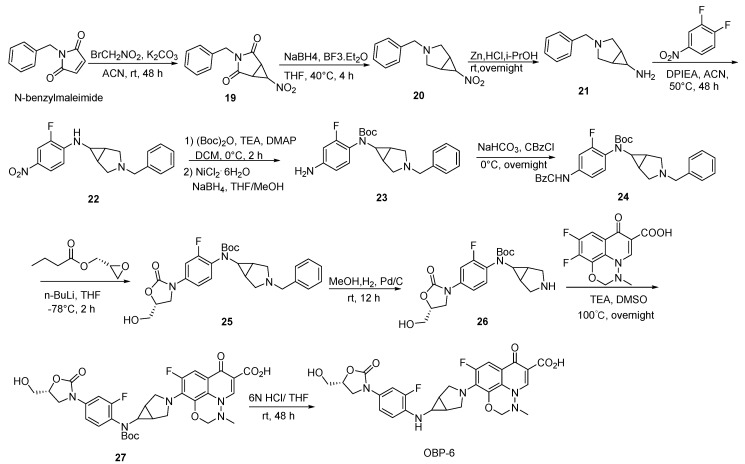
Synthesis of hybrid OBP-6.

**Scheme 7:**
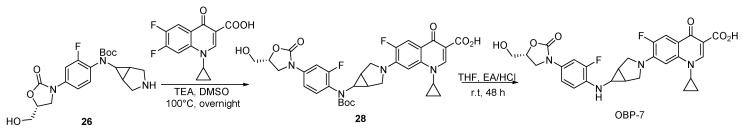
Synthesis of hybrid OBP-7.

The synthesis of oxazolidinone-fluoroquinolone hybrids OBP-(1–7) was performed as outlined in Scheme 1, Scheme 2, Scheme 3, Scheme 4, Scheme 5, Scheme 6 and Scheme 7. The hybrids OBP-(1–5) consisted of two parts, compound 8 and corresponding acids (Scheme 2, Scheme 3, Scheme 4 and Scheme 5). Compound 8 was synthesized according to the following Scheme 1. The corresponding acids of OBP-1 and OBP-2 were obtained from commercial sources, and those of OBP-(3–5) were synthesized by another routes (Scheme 3, Scheme 4 and Scheme 5). Meanwhile, we also prepared hybrids OBP-6 and OBP-7, containing an exo-3-methyl-6-amino-3-azabicyclo [3.1.0] hexane linker between the oxazolidinone and fluoroquinolone elements (Scheme 6 and Scheme 7). The structures of the target hybrids OBP-(1–7) were further characterized (Supplementary Materials).

### 2.2. Antibacterial Activities

#### 2.2.1. In Vitro Activities of Oxazolidinone-Fluoroquinolone Hybrids

The in vitro antibacterial activities of oxazolidinone-fluoroquinolone hybrids against a panel of well-characterized susceptible and resistant Gram-positive and Gram-negative bacteria were evaluated and the results were presented as MICs expressed in µg/mL (Table 1). CZD (cadazolid), LZD (linezolid) and moxifloxacin (MXF) were used as comparator drugs. As shown in Table 1, MIC values of LZD and MXF against standard strains were within the acceptable range, indicating that the test results were reliable. 

For Gram-positive bacteria, most oxazolidinone-fluoroquinolone hybrids with the exceptions of OBP-6 and OBP-7 exhibited the potent antibacterial activities with MICs ranging from 0.031 µg/mL to 2 µg/mL, even against levofloxacin-resistant *Streptococcus agalactiae* (*S. agalactiae*), vancomycin-resistant *enterococcus* (VRE) and methicillin-resistant *Staphylococcus aureus* (MRSA). Among these new hybrids, OBP-4 was the most active compound against Gram-positive bacteria (MICs ≤ 0.5 µg/mL), which was twice as active overall than the comparator agent CZD, a oxazolidinone-fluoroquinolone hybrid in phase III clinical trials [23]. The antibacterial efficacy of hybrid OBP-5 against Gram-positive bacteria was comparable to that of CZD, but was approximately 4–16 times stronger than that of LZD. The antibacterial activities of hybrids OBP-1, OBP-2 and OBP-3 against Gram-positive bacteria laid between that of CZD and LZD. Gram-positive bacteria were less susceptible to OBP-6 and OBP-7 than other hybrids, especially resistant strains. For Gram-negative bacteria, only *Haemophilus influenzae* (*H. influenzae*) was susceptible to these hybrids, while hybrids OBP-6 and OBP-7 also exhibited good activities against *Escherichia coli* (*E. coli*) (Table 1). The above results demonstrated that the antibacterial activities of these oxazolidinone-fluoroquinolone hybrids were mainly restricted to Gram-positive bacteria, suggesting a more pronounced oxazolidinone-type antibacterial spectrum. 

Obviously, hybrids OBP-6 and OBP-7 differed greatly from other hybrids in antibacterial activities and spacer, which indicated the spacer was a major factor influencing their antibacterial activities [20]. Meanwhile, the antibacterial activity was more or less changed due to the differences of the substituents at *N*^1^-position of fluoroquinolone pharmacophores.

#### 2.2.2. In Vitro Activities of Hybrids OBP-4 and OBP-5 against Clinical Isolates

As can be seen from the above data, OBP-4 and OBP-5 were the two most active oxazolidinone-fluoroquinolone hybrids. Therefore, the susceptibility of OBP-4 and OBP-5 against 263 clinical isolates were further tested. The resulting MICs for OBP-4, OBP-5 and comparator drugs enrofloxacin (ENR), ciprofloxacin (CIP), gatifloxacin (GAFX), nadifloxacin (NAFX), LZD and vancomycin (VAN) were listed in Table 2. For Gram-positive isolates, hybrids OBP-4 and OBP-5 had the lowest MICs among all tested compounds, with MIC_90_ values of 0.125–0.25 µg/mL for OBP-4 and ≤0.0625–0.25 µg/mL for OBP-5. For Gram-negative isolates (*Haemophilus parasuis*), hybrids OBP-4 and OBP-5 had relatively weak activities with MIC_90_ values of 4 µg/mL and 2 µg/mL, respectively (Table 2). 

For *Staphylococcus aureus* (*S. aureus*), *Staphylococcus haemolyticus* (*S. haemolyticus*), *Staphylococcus epidermidis* (*S. epidermidis*) and *Streptococcus suis* (*S. suis*), most clinical isolates (>90%) can be inhibited by hybrids OBP-4 and OBP-5 at 0.25 µg/mL, and MIC_90_ values of OBP-4 and OBP-5 against these isolates were much lower than those of comparator agents (Table 2 and Table 3). Against *Streptococcus pneumoniae* (*S. pneumoniae*) isolates, OBP-4 and OBP-5 retained the excellent activities with MIC_90_ of 0.25 µg/mL, while comparator drugs were almost inactive with MIC_90_ ≥ 32 µg/mL except for LZD (Table 2). Furthermore, OBP-4 and OBP-5 showed 2- to 16-fold higher activity against *Enterococcus faecalis* (*E. faecalis*), and 16- to 128-fold higher activity against *Enterococcus faecium* (*E. faecium*) than comparator agents (Table 2). Although OBP-4 and OBP-5 were less active against *H. parasuis* than quinolone antimicrobials, they were much better than LZD and VAN (Table 2). Therefore, hybrids OBP-4 and OBP-5 were the most potent compounds against tested clinical isolates. Overall, OBP-5 exhibited 2-fold greater activity than OBP-4, which can be best explained by the difference of the substituents at *N*^1^-position of fluoroquinolone pharmacophores between OBP-4 and OBP-5. 

Meanwhile, hybrids OBP-4 and OBP-5 were also tested against a group of common resistant strains, including MRSA, methicillin-resistant *S. epidermidis* (MRSE), CIP-resistant *S. aureus*, CIP-resistant *S. haemolyticus*, VAN- and quinolone-resistant *S. pneumoniae*, LZD-resistant *E. faecalis*, and LZD or VAN- and quinolone-resistant *E. faecium*. The activities of OBP-4 and OBP-5 against MRSA and MRSE were equivalent to those of quinolone comparator agents ENR, CIP, GAFX and NDFX, but markedly superior to those of LZD and VAN (Table 4). For other resistant organisms in the test, MICs values of OBP-4 and OBP-5 ranged from ≤ 0.0625 to 1 µg/mL, and were much lower than the corresponding MICs values of comparator agents (Table 4). Similarly, OBP-5 displayed higher activities against tested resistant strains than OBP-4. The superior activities of OBP-4 and OBP-5 against resistant strains indicated that they had no cross-resistance with tested oxazolidinones or quinolones.

Again, the superior antibacterial activities of hybrids OBP-4 and OBP-5 against Gram-positive bacteria including drug-resistant strains were confirmed. The result hinted that OBP-4 and OBP-5 may have a similar mode of action with oxazolidinones. In this study, the reduced membrane- permeability may be a leading cause of poor activities of OBP-4 and OBP-5 against Gram-negative bacteria. Intrinsically, the weak fluoroquinolone-like characteristic of OBP-4 and OBP-5 could be attributed to the large molecular volume and decreased liposolubility caused by the hybridization.

### 2.3. Mode of Action of Hybrids OBP-4 and OBP-5

#### 2.3.1. In Vitro Enzyme Assays of Hybrids OBP-4 and OBP-5

**Figure 1:**
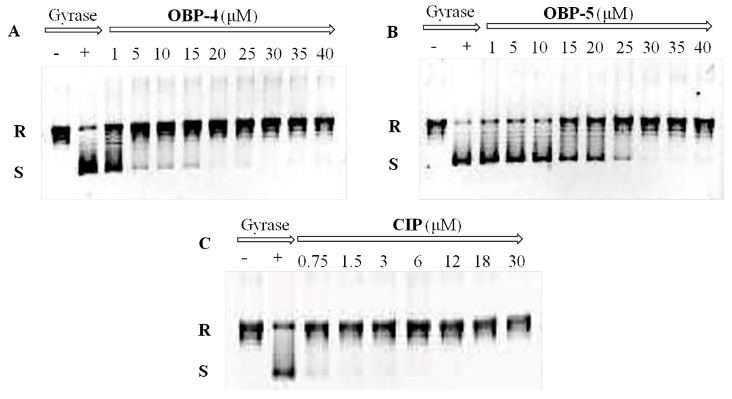
Inhibition assay of OBP-4 (**A**), OBP-5 (**B**) and CIP (**C**) on DNA gyrase. Line 1(−): no compound and DNA gyrase; Line 2(+): DNA gyrase present and compound absent; Line 3-11(9): DNA gyrase with different concentrations of tested compound. R: relaxed DNA; S: supercoiled DNA.

**Figure 2:**
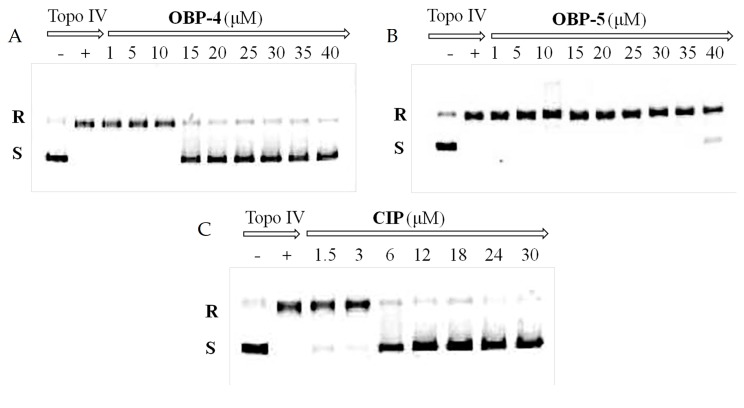
Inhibition assay of OBP-4 (**A**), OBP-5 (**B**) and CIP (**C**) on Topo IV. Line 1(−): no compound and Topo IV; Line 2(+): Topo IV present and compound absent; Line 3-11(9): Topo IV with different concentrations of tested compound. R: relaxed DNA; S: supercoiled DNA.

In order to explore the mode of action of OBP-4 and OBP-5, we carried out the enzyme assays for these two hybrids. The results were presented in Figure 1 and Figure 2 and Table 5.

DNA gyrase and topoisomerase IV (Topo IV), two well-known targets for quinolone antimicrobials [24], play a vital role in the replication and transcription of DNA [25]. Therefore, the impact of OBP-4 and OBP-5 on DNA gyrase and topo IV was firstly investigated by DNA gyrase supercoiling assay and Topo IV relaxation assay, respectively. As shown in Figure 1, OBP-4 and OBP-5 could inhibit DNA gyrase in a dose-dependent manner with IC_50_ values being 1–5 μM and 20 μM for OBP-4 and OBP-5, respectively (Table 5). By comparison, the inhibition action of OBP-4 and OBP-5 on DNA gyrase was evidently inferior to the reference drug CIP (IC_50_, <0.75 μM). Similar results but with higher IC_50_ values were found for hybrids OBP-4 and OBP-5 in the topo IV relaxation assay. OBP-4 (IC_50_, 10–15 μM) was less active in inhibiting topo IV compared with CIP (IC_50_, 3–6 μM) while OBP-5 was the least active (IC_50_ > 40 μM) (Figure 2, Table 5). Overall, hybrid OBP-4 exhibited a much better inhibitory activity on DNA synthesis than OBP-5, but they were significantly less active than CIP. 

Oxazolidinones have a unique mechanism of action involving inhibition of protein synthesis by binding the ribosomal 50S subunit [16]. Thus, the action of hybrids OBP-4, OBP-5 and the reference drug LZD on protein synthesis was investigated with the *E. coli*-based cell-free protein synthesis system. As expected, OBP-4 and OBP-5 showed potent inhibition of protein synthesis in vitro transcription/translation assay. IC_50_ values of OBP-4 and OBP-5 were 5 μM for OBP-4 and 2 μM for OBP-5, respectively (Table 5), which were equal or 2.5-fold greater than that of LZD (IC_50_, 5 μM) (Table 5). 

The above data demonstrated that the protein synthesis inhibition was the primary mode of action for OBP-4 and OBP-5 whilst the activity against DNA synthesis was weak, which correlated well with the observed antibacterial activity pattern. The results confirmed our previous speculation. For the imbalanced dual-action mode, the possible explanation was that the introduction of oxazolidinone substructure or spacer in hybrid changed the intrinsic binding affinity of fluoroquinolone in two targets.

#### 2.3.2. Molecular Docking of Hybrids OBP-4 and OBP-5

**Figure 3:**
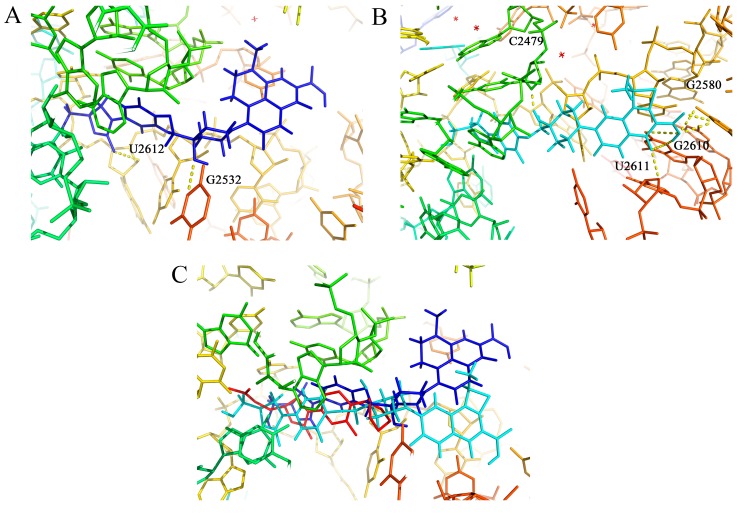
(**A**) The optimal docking conformation of OBP-4 (blue sticks) in the active site of 50S subunit (PDB ID: 4WFA). (**B**) The optimal docking conformation of OBP-5 (light blue sticks) in the active site of 50S subunit (PDB ID: 4WFA). (**C**) The superposition of OBP-4, OBP-5 and LZD (red sticks) in the active site of 50S subunit (PDB ID: 4WFA). Hydrogen bonds are showed as dashed lines.

As shown in Table 5, OBP-4 and OBP-5 displayed a strong inhibition action in protein synthesis. To understand the binding mode of OBP-4 and OBP-5, the molecular docking was performed between OBP-4 (or OBP-5) and the active site of 50S subunit. The optimal docking conformations of tested compounds in the active site of 50S subunit were presented in Figure 3. The blue, light blue and red sticks represented the optimal conformations of OBP-4 (Figure 3A), OBP-5 (Figure 3B) and LZD (Figure 3C). As expected, both OBP-4 and OBP-5 can bind to the active site of the 50S subunit. OBP-4 were docked into the active site of 50S subunit, with its oxazolidinone part forming hydrogen bonds with U-2612 and G-2532 (Figure 3A), while OBP-5 were surrounded by G-2580, G-2610, U-2611 and C-2479 (Figure 3B). Furthermore, OBP-4, OBP-5 and LZD have a similarity in the optimal conformations and binding modes to the active site of 50S subunit (Figure 3C). This similarity could explain why OBP-4 and OBP-5 have an oxazolidinone-like antibacterial spectrum and a strong inhibitory activity on protein synthesis. Meanwhile, the better antibacterial activities of OBP-4 and OBP-5 than that of LZD may be attributed to the interactions between fluoroquinolone pharmacophores and the 50S subunit. The further investigations are required to clearly elucidate the mode of action of OBP-4 and OBP-5.

### 2.4. Acute Toxicity Test

The previous study has shown that CZD, structurally similar to hybrids OBP-4 and OBP-5, has a good safety level [26]. Therefore, the oral acute toxicity of hybrids OBP-4 and OBP-5 was directly evaluated with a limit test dose of 2000 mg/kg. No death and any toxicity signs were observed for all animals until the experiment was finished. The oral LD_50_ values for hybrids OBP-4 and OBP-5 were considered to be higher than 2000 mg/kg, suggesting that hybrids OBP-4 and OBP-5 are likely to be safe for future application, at least with a single oral dose.

## 3. Materials and Methods 

### 3.1. General

The chemicals and reagents were acquired commercially and used without further purification. Reactions were monitored by thin-layer chromatography (TLC) plates pre-coated with 0.2-mm-thick silica gel GF254 (Qingdao Ocean Chemical Co., Ltd., Shandong, China). The visualized analysis of products was achieved by UV illumination at 254 nm and staining in the solution of bismuth nitrate and 0.05% KMnO_4_. The determination of the melting point was conducted with an X-4 microscopic melting point apparatus (Beijing Tech Instrument Co., Ltd., Beijing, China). ^1^H-NMR and ^13^C-NMR were performed on a Bruker spectrometer (Bruker BioSpin, Zürich, Switzerland) at 400 and 151 MHz, respectively. All NMR spectra were reported in chemical shifts (δ, ppm) with residual solvents as internal standards. Multiplicities were expressed as s (singlet), d (doublet), dd (doublet of doublets), t (triplet) and m(multiplet), and coupling constants were expressed as *J* in hertz (Hz). Electrospray ionization mass spectra (ESI-MS) were recorded with a Shimadzu mass spectrometer (Shimadzu, Tokyo, Japan) and high resolution mass spectra (HRMS) were obtained with an Agilent Q-TOF 6530 mass spectrometer (Agilent Technologies, Palo Alto, CA, USA).

### 3.2. Chemistry

#### 3.2.1. Preparation of Hybrids OBP-1, OBP-2 and OBP-3

Compound **1**: a mixture of 2-fluoro-4-nitrophenol (10.00 g, 63.69 mmol, 1 eq) [27], benzyl bromide (11.98 g, 70.00 mmol, 1.1 eq), K_2_CO_3_ (13.18 g, 95.54 mmol, 1.5 eq) and KI (1.06 g, 6.37 mmol) were added to acetone (0.10 L) and then heated to 60 °C overnight. Upon cooling to room temperature and addition of ice water, yellow solid was precipitated and dried (15.00 g, 95%), m.p., 197–198 °C. ^1^H-NMR (400 MHz, DMSO-*d*_6_) δ 8.18 (dd, *J* = 11.2, 2.8 Hz, 1H), 8.13 (d, *J* = 9.2 Hz, 1H), 7.53–7.39 (m, 6H), 5.35 (s, 2H). ESI-MS (*m*/*z*): 247.9 [M + H]^+^.

Compound **2**: compound **1** (72.80 g, 294.74 mmol, 1 eq), reduced iron powder (99.00 g, 1768.42 mmol, 6 eq) and ammonium chloride (94.59 g, 1768.42 mmol, 6 eq) were mixed with EtOH:H_2_O (2:1), and refluxed at 90 °C for 2 h. After cooling to room temperature, the mixture was filtered. The filtered and concentrated to give the brown oily substance compound **2** (60.00 g, 94%). ^1^H-NMR (400 MHz, CDCl_3_) δ 7.42 (d, *J* = 7.2 Hz, 2H), 7.36 (t, *J* = 7.2 Hz, 2H), 7.31 (t, *J* = 7.0 Hz, 1H), 6.80 (t, *J* = 9.0 Hz, 1H), 6.46 (dd, *J* = 12.4, 2.8 Hz, 1H), 6.33 (d, *J* = 8.8 Hz, 1H), 5.03 (s, 2H). ESI-MS (*m*/*z*): 217.9 [M + H]^+^.

Compound **3**: a saturated sodium bicarbonate solution (0.50 L) was added to compound **2** (60.00 g, 276.50 mmol, 1 eq) in anhydrous THF (0.50 L) and cooled to 0 °C. The mixed solution was treated dropwise with benzyl carbonochloridate (51.88 g, 304.15 mmol, 1.1 eq) and then stirred for 30 min at 0 °C. The mixture was extracted with EtOAc (3.00 L*2) and brine (2.40 L). The organic layer was dried (Na_2_SO_4_), filtered, and concentrated in vacuo to give compound **3** (88.00 g, 91%) as a yellow solid, m.p., 116–118 °C. ^1^H-NMR (400 MHz, CDCl_3_) δ 7.48–7.27 (m, 11H), 6.95–6.87 (m, 2H), 6.57 (s, 1H), 5.19 (s, 2H), 5.10 (s, 2H). ESI-MS (*m*/*z*): 351.9 [M + H]^+^.

Compound **4**: the suspension of compound **3** (40.00 g, 113.96 mmol, 1 eq) in THF (1.00 L) was cooled to −78 °C, and then n-BuLi (2.50 M in hexane 54.70 mL, 136.75 mmol, 1.2 eq) was added dropwisely and stirred at −78 °C for 0.5 h. Subsequently, (*R*)-oxiran-2-ylmethyl butyrate (20.35 g, 141.31 mmol, 1.24 eq) in THF (0.50 L) was added dropwisely to the mixed solution within 30 min. The reaction mixture was warmed up slowly and stirred at room temperature overnight. The mixture was extracted with EtOAc (2.00 L*2) and brine (1.50 L). The organic layer was dried (MgSO_4_), filtered and concentrated in vacuo to give compound **4** (31.50 g, 87%) as a yellow solid, m.p., 135–137 °C. ^1^H-NMR (400 MHz, DMSO-*d*_6_) δ 7.58 (dd, *J* = 13.6, 2.4 Hz, 1H), 7.44 (d, *J* = 7.2 Hz, 2H), 7.39 (t, *J* = 7.4 Hz, 2H), 7.33 (t, *J* = 6.8 Hz, 1H), 7.26 (t, *J* = 9.2 Hz, 1H), 7.22–7.16 (m, 1H), 5.22 (t, *J* = 5.6 Hz, 1H), 5.16 (s, 2H), 4.67 (dd, *J* = 9.2, 5.6 Hz, 1H), 4.03 (t, *J* = 9.0 Hz, 1H), 3.78 (dd, *J* = 8.8, 6.4 Hz, 1H), 3.69–3.62 (m, 1H), 3.58–3.50 (m, 1H). ESI-MS (*m*/*z*): 317.9 [M + H]^+^.

Compound **5**: Pd/C (1.50 g, 5%) was carefully added to a solution of compound **4** (31.00 g, 97.79 mmol, 1 eq) in MeOH (0.04 L). The reaction was proceeded with H_2_ at room temperature for 12 h. The liquid was filtered, concentrated and purified by recrystallization in petroleum ether and ethyl acetate (2:1) to give compound **5** (21.76 g, 98%) as a white solid, m.p., 179–181 °C. ^1^H-NMR (400 MHz, DMSO-*d*_6_) δ 9.67 (s, 1H), 7.47 (d, *J* = 13.6 Hz, 1H), 7.09 (d, *J* = 8.8 Hz, 1H), 6.95 (t, *J* = 9.4 Hz, 1H), 5.20 (s, 1H), 4.65 (s, 1H), 4.01 (t, *J* = 9.0 Hz, 1H), 3.76 (t, *J* = 7.4 Hz, 1H), 3.65 (d, *J* = 11.6 Hz, 1H), 3.54 (d, *J* = 12.4 Hz, 1H). ESI-MS (*m*/*z*): 227.9 [M + H]^+^.

Compound **6**: the sodium hydroxide (60%, 4.37 g, 109.25 mmol, 1.5 eq) was added to a solution of trimethylsulfoxonium iodide (24.06 g, 109.32 mmol, 1.5 eq) in dry DMSO (0.10 L) at 0 °C, and then stirred at 0 °C for 40 min. Benzyl 4-oxopiperidine-1-carboxylate (17.00 g, 72.88 mmol, 1 eq) [28] was added to the mixture and stirred for 2 h at 55 °C. The reaction mixture was extracted with EtOAc (0.18 L) and brine (0.06 L). The organic layer was dried (Na_2_SO_4_) and concentrated in vacuo. Crude was purified by flash column chromatography (hexanes/EtOAc) on silica gel column to give compound **6** (14.50 g, 80%) as a yield oil. ^1^H-NMR (400 MHz, CDCl_3_) δ 7.42–7.28 (m, 5H), 5.15 (s, 2H), 3.82 (s, 2H), 3.48 (t, *J* = 10.8 Hz, 2H), 2.70 (s, 2H), 1.83 (s, 2H), 1.45 (d, *J* = 10.4 Hz, 2H). ESI-MS (*m*/*z*): 247.9 [M + H]^+^.

Compound **7**: the mixed solution of compound **5** (8.00 g, 35.24 mmol, 1 eq) and compound **6** (13.06 g, 52.86 mmol, 1.5 eq) DMF (0.05 L) was treated with Na_2_CO_3_ (7.47 g, 70.48 mmol, 2 eq). The resulting solution was heated to 100 °C and stirred for 12 h. The reaction was quenched by H_2_O and extracted by EtOAc (0.20 L*3). The combined organic layer was washed by brine and dried with anhydrous Na_2_SO_4_. After evaporation and recrystallization, compound **7** (17.00 g, 90%) was obtained as a white solid, m.p., 121–123 °C. ^1^H-NMR (400 MHz, DMSO-*d*_6_) δ 7.61–7.54 (m, 1H), 7.40–7.30 (m, 5H), 7.23–7.16 (m, 2H), 5.21 (t, *J* = 5.6 Hz, 1H), 5.08 (s, 2H), 4.84 (s, 1H), 4.68 (dd, *J* = 9.2, 5.6 Hz, 1H), 4.04 (t, *J* = 9.0 Hz, 1H), 3.87–3.81 (m, 4H), 3.81–3.76 (m, 1H), 3.70–3.62 (m, 1H), 3.60–3.49 (m, 1H), 3.17 (d, *J* = 3.6 Hz, 2H), 1.66–1.50 (m, 4H). ESI-MS (*m*/*z*): 475.0 [M + H]^+^.

Compound **8**: Pd/C (1.00 g, 5%) was carefully added to a solution of compound **7** (17.00 g, 35.86 mmol, 1 eq) in MeOH (0.50 L). The reaction was proceeded with H_2_ at room temperature for 12 h. The mixture was filtered, concentrated and purified by recrystallization to obtained compound **8** as a white solid (11.00 g, 92%), m.p., 179–181 °C. ^1^H-NMR (400 MHz, DMSO-*d*_6_) δ 7.57 (d, *J* = 13.2 Hz, 1H), 7.25–7.12 (m, 2H), 5.24 (s, 1H), 4.70–4.65 (m, 1H), 4.52 (s, 1H), 4.04 (t, *J* = 9.0 Hz, 1H), 3.85–3.73 (m, 3H), 3.66 (dd, *J* = 12.4, 3.2 Hz, 1H), 3.55 (dd, *J* = 12.4, 4.0 Hz, 1H), 2.81 (t, *J* = 10.6 Hz, 2H), 2.67 (d, *J* = 12.0 Hz, 2H), 1.65–1.51 (m, 2H), 1.44 (d, *J* = 12.8 Hz, 2H). ESI-MS (*m*/*z*): 340.9 [M + H]^+^.

Compound **9**: to a solution of (S)-9,10-difluoro-2,3-dihydro-3-methyl-7-oxo-7H-pyrido[1,2,3-de] -1,4-benzoxazine-6-carboxylic acid (1.00 g, 3.56 mmol, 1 eq) in H_2_SO_4_ (98%, 0.02 L) [29], KNO_3_ (0.719 g, 7.12 mmol, 2 eq) was added and then stirred at 0 °C for 30 min. The reaction mixture was diluted with ice water (0.05 L) and filtered. Compound **9** Yield (1.00 g, 86%) was obtained as a yellow solid. ^1^H-NMR (400 MHz, DMSO-*d*_6_) δ 9.15 (s, 1H), 5.08 (d, *J* = 6.8 Hz, 1H), 4.76 (d, *J* = 10.8 Hz, 1H), 4.51 (d, *J* = 9.6 Hz, 1H), 1.47 (d, *J* = 6.8 Hz, 3H).

Compound **10**: Pd/C (0.05 g, 5%) was carefully added to a solution of compound **9** (1.00 g, 3.07 mmol, 1 eq) in MeOH (0.05 L). The reaction proceeded with H_2_ at room temperature for 12 h. The liquid was filtered and concentrated to give compound **10** (0.50 g, 55%) a yellow oil. ^1^H-NMR (400 MHz, DMSO-*d*_6_) δ 8.86 (s, 1H), 4.86 (d, *J* = 6.8 Hz, 1H), 4.47 (d, *J* = 11.2 Hz, 1H), 4.21 (d, *J* = 10.4 Hz, 1H), 1.40 (d, *J* = 6.8 Hz, 3H). ESI-MS (*m*/*z*): 300.9 [M + H]^+^.

General procedure for the synthesis of hybrids OBP-1, OBP-2 and OBP-3.

A suspension of compound **8** (1.1 eq), TEA (5 eq) and corresponding acid (1 eq) in DMSO were mixed and stirred at 120 °C for 20 h. The mixed solution was cooled to room temperature, adjusted pH (<6) with 50% H_2_SO_4_, filtered and concentrated in vacuo. Finally, the target hybrids were obtained by recrystallization in petroleum ether and ethyl acetate (2:1).

OBP-1: while solid (0.516 g, 30%), m.p., 256–257 °C. ^1^H-NMR (400 MHz, DMSO-*d*_6_) δ 15.11 (s, 1H), 8.76 (s, 1H), 7.66–7.53 (m, 2H), 7.21 (dd, *J* = 14.4, 5.2 Hz, 2H), 5.32 (s, 2H), 5.22 (s, 1H), 4.82 (s, 1H), 4.71–4.66 (m, 1H), 4.05 (t, *J* = 8.8 Hz, 1H), 3.88 (s, 2H), 3.83–3.77 (m, 1H), 3.67 (d, *J* = 12.8 Hz, 1H), 3.57–3.50 (m, 3H), 3.23 (s, 2H), 3.02 (s, 3H), 1.88 (t, *J* = 11.0 Hz, 2H), 1.65 (d, *J* = 12.8 Hz, 2H).^13^C-NMR (151 MHz, DMSO-*d*_6_) δ 176.50 (d, *J* = 3.02 Hz), 165.98, 155.89 (d, *J* = 246.13 Hz), 154.88, 151.85 (d, *J* = 226.50 Hz), 145.73, 143.15 (d, *J* = 10.57 Hz), 138.54 (d, *J* = 7.55 Hz), 132.92 (d, *J* = 15.1 Hz), 132.74, 124.37, 119.51 (d, *J* = 9.06 Hz), 116.35, 114.27, 107.06 (d, *J* = 22.65 Hz), 106.69, 104.21 (d, *J* = 24.16 Hz), 83.22, 78.01, 73.57, 67.56, 62.25, 46.61, 46.51, 43.31, 34.46. ESI-MS (*m*/*z*) [M + H]^+^ calcd. for C_28_H_28_F_2_N_4_O_9_: 603.1897, found: 603.1906.

OBP-2: while solid (0.60 g, 56%), m.p., 269–270 °C. ^1^H-NMR (400 MHz, DMSO-*d*_6_) δ 15.26 (s, 1H), 8.96 (s, 1H), 7.58 (d, *J* = 12.4 Hz, 2H), 7.27–7.18 (m, 2H), 5.22 (t, *J* = 5.6 Hz, 1H), 4.97–4.86 (m, 1H), 4.79 (s, 1H), 4.72–4.65 (m, 1H), 4.59 (d, *J* = 10.8 Hz, 1H), 4.39 (s, 1H), 4.05 (t, *J* = 9.0 Hz, 1H), 3.88 (s, 2H), 3.80 (dd, *J* = 8.8, 6.4 Hz, 1H), 3.72–3.62 (m, 1H), 3.58–3.46 (m, 3H), 3.24 (d, *J* = 11.6 Hz, 2H), 1.96–1.79 (m, 2H), 1.64 (d, *J* = 12.8 Hz, 2H), 1.45 (d, *J* = 6.8 Hz, 3H). ^13^C-NMR (151 MHz, DMSO-*d*_6_) δ 176.80 (d, *J* = 3.02 Hz), 166.52, 156.00 (d, *J* = 247.64 Hz), 154.88, 151.90 (d, *J* = 243.11 Hz), 146.51, 143.16 (d, *J* = 12.08 Hz), 140.49 (d, *J* = 6.04 Hz), 133.28 (d, *J* = 15.1Hz), 132.73, 125.27, 119.76 (d, *J* = 9.06 Hz), 116.34, 114.26, 107.05 (d, *J* = 24.16 Hz), 107.01, 103.70 (d, *J* = 24.16 Hz), 77.67, 73.57, 68.44, 68.15, 62.07, 55.26, 46.61, 46.55, 34.48, 18.36.ESI-MS (*m*/*z*) [M + H]^+^ calcd. for C_2__9_H_29_F_2_N_3_O_9_: 602.1945, found: 602.1948.

OBP-3: yellow solid (0.70 g, 83%), m.p., 255–256 °C. ^1^H-NMR (400 MHz, DMSO-*d*_6_) δ 15.09 (s, 1H), 8.68 (s, 1H), 7.59 (d, *J* = 14.4 Hz, 1H), 7.29–7.12 (m, 2H), 6.91 (s, 2H), 5.23 (s, 1H), 4.82–4.61 (m, 3H), 4.38 (d, *J* = 11.2 Hz, 1H), 4.16–3.99 (m, 2H), 3.87 (s, 2H), 3.83–3.76 (m, 1H), 3.67 (d, *J* = 11.2 Hz, 1H), 3.55 (d, *J* = 14.4 Hz, 1H), 3.50–3.42 (m, 2H), 3.24 (s, 2H), 1.93–1.75 (m, 2H), 1.63 (d, *J* = 12.0 Hz, 2H), 1.38 (d, *J* = 6.4 Hz, 3H). ^13^C-NMR (151 MHz, DMSO-*d*_6_) δ 180.25 (d, *J* = 3.02 Hz), 166.62, 154.89, 151.90 (d, *J* = 243.11 Hz), 146.78, 144.42, 143.17 (d, *J* = 12.08 Hz), 141.26 (d, *J* = 235.56 Hz), 134.31 (d, *J* = 15.1 Hz), 132.68 (d, *J* = 9.06 Hz), 127.91 (d, *J* = 6.04 Hz), 126.15, 124.09, 116.34 (d, *J* = 1.51 Hz), 114.25, 109.99, 107.06 (d, *J* = 24.16 Hz), 105.67, 77.66, 73.57, 68.22, 67.23, 62.06, 55.74, 46.61, 34.53, 18.31. ESI-MS (*m*/*z*) [M + H]^+^ calcd. for C_2__9_H_30_F_2_N_4_O_9_: 617.2054, found: 617.2063.

#### 3.2.2. Preparation of Hybrid OBP-4

Compound **11**: to a stirred solution of H_2_SO_4_ (7.78 mL) in Ac_2_O (0.778 L, 8.21 mol), 3,4-difluoroaniline (500.00 g, 3.88 mol) [30] was added at about 10 °C, and then stirred at room temperature for 30 min. The mixture was poured into ice water, filtered, the filter cake was washed with water and dried to give an off-white solid (604.00 g, 91%). ^1^H-NMR (400 MHz, CDCl_3_) δ 7.63–7.56 (m, 1H), 7.53 (s, 1H), 7.12–7.03 (m, 2H), 2.17 (s, 3H). ESI-MS (*m*/*z*): 171.9 [M + H]^+^.

Compound **12**: to a stirred solution of compound **11** (588.00 g, 3.44 mol), NaOAc (338.00 g, 4.12 mol) in AcOH (1.94 L) was added dropwise to a solution of Br_2_ (0.212 L, 4.12 mol) in AcOH (0.60 L) at 60 °C, then the mixture was stirred at 80 °C until SM was disappeared. After cooling to room temperature, the mixture was poured into ice water, filtered, the filter cake was washed with water and dried to give a white solid (735.00 g, 85%). ^1^H-NMR (400 MHz, CDCl_3_) δ 8.40–8.30 (m, 1H), 7.60–7.48 (m, 1H), 7.41–7.35 (m, 1H), 2.24 (s, 3H). ESI-MS (*m*/*z*): 249.8 [M + H]^+^.

Compound **13**: a mixture of compound **12** (565.00 g, 2.26 mol), sodium m-nitrobenzensulfonate (508.00 g, 2.26 mol), iron sulfate heptahydrate (63.30 g, 158.00 mmol), boric acid (616.00 g, 9.94 mol), water (2.16 L) and concentrated HCl (2.16 L) was refluxed for 0.5 h. Crotonaldehyde (0.278 L, 3.38 mol) was added dropwise to the mixture at the same temperature and stirring was continued for 1 h under reflux. After cooling to room temperature, the insoluble matter was filtered off and the filtrate was poured into MeOH (0.80 L). The solution was neutralized with 25% (*w*/*v*) aqueous NaOH, cooled at ice bath. The resultant precipitates were collected by filtration, the wet solid was dissolved in DCM (2.00 L) and dried over anhydrous sodium sulfate, filtered, the filtrate was concentrated in vacuo and the crude product was recrystallized from hexane to give an off-white solid (330.00 g, 57%). ^1^H-NMR (400 MHz, CDCl_3_) δ 8.31 (d, *J* = 8.4 Hz, 1H), 7.94–7.88 (m, 1H), 7.42 (d, *J* = 8.8 Hz, 1H), 2.82 (s, 3H).

Compound **14**: a mixture of compound **13** (330.00 g, 1.28 mol), 10% wet Pd/C (6.60 g), and sodium acetate (105.00 g, 1.28 mol) in 90% (*v*/*v*) aqueous AcOH (2.00 L) was hydrogenated at 50 °C under a hydrogen pressure 1–5 atm. After the hydrogen absorption had ceased, the catalyst was filtered off and the filtrate was concentrated under reduced pressure until about half of the solvent was evaporated, the residue was poured into water, the resulting precipitates were collected by filtration and dried to give a light red solid as the first crop (160.00 g), the filtrate was extracted with EA, the combined organic layer was washed with saturated aqueous NaHCO_3_, dried over Na_2_SO_4_ and evaporated in vacuo to give a brown solid as the second crop (65.00 g).The combined crops (225.00 g, 1.26 mol) were dissolved in AcOH (1.10 L), to the stirred solution was added NaBH_3_CN (198.00 g, 3.15 mol), then the mixture was stirred at room temperature for 6 h, to the mixture was added saturated aqueous Na_2_CO_3_ until there was no bubble produced, extracted with EA and the combined organic layer was washed with brine, dried over anhydrous sodium sulfate and concentrated in vacuo, the residue was purified by silica gel column chromatography (1% EA in PE) to give a colorless oil (180.00 g, 77%). ^1^H-NMR (400 MHz, CDCl_3_) δ 6.80–6.71 (m, 1H), 6.18–6.09 (m, 1H), 3.64 (brs, 1H), 3.35–3.26 (m, 1H), 2.90–2.80 (m, 1H), 2.70–2.58 (m, 1H), 1.99–1.90 (m, 1H), 1.56–1.44 (m, 1H), 1.20 (d, *J* = 6.0 Hz, 3H). ESI-MS (*m*/*z*): 184.0 [M + H]^+^.

Compound **15**: compound **14** (174.00 g, 951.00 mmol) was added to a stirred solution of L-DBTA (187.00 g, 522.00 mmol) in EA (0.35 L) and the mixture was stirred at room temperature for 2 h. The resulting precipitates were collected by filtration and recrystallized from 60% (*v*/*v*) aqueous MeOH. The resulting solid was treated with aqueous NaOH to give a colorless oil (58.00 g, 33%), 100% ee as determined by chiral HPLC. ^1^H-NMR (400 MHz, CDCl_3_) δ 6.80–6.71 (m, 1H), 6.18–6.09 (m, 1H), 3.67 (s, 1H), 3.35- 3.26 (m, 1H), 2.90–2.80 (m, 1H), 2.70–2.58 (m, 1H), 1.99–1.90 (m, 1H), 1.56- 1.44 (m, 1H), 1.20 (d, *J* = 6.4 Hz, 3H).

Compound **16**: A mixture of compound **15** (58.00 g, 317.00 mmol) and EMME (103.00 g, 477.00 mmol) was heated at 130 °C with stirring for 3 h and the reaction mixture was added dropwise to the pre-heated PPA (161.00 g) at 130 °C. When the addition was complete, the mixture was stirred at the same temperature for 1 h. Then EtOH (0.875 L), concentrated HCl (0.058 L) and water (0.58 L) were added, the mixture was refluxed for 1 h. After cooling, water (1.13 L) was added to the mixture and the resulting precipitates were collected by filtration, the solid was washed with water then ether and dried to give an off-white solid (62.00 g, 70%). ^1^H-NMR (400 MHz, DMSO-*d*_6_)) δ 14.96 (s, 1H), 9.06 (s, 1H), 8.13–8.06 (m, 1H), 5.02–4.89 (m, 1H), 3.16–3.08 (m, 1H), 3.07–2.95 (m, 1H), 2.23–2.07 (m, 2H), 1.39 (d, *J* = 6.8 Hz, 3H). ESI-MS (*m*/*z*): 279.9 [M + H]^+^.

Compound **17**: A mixture of boric anhydride (2.74 g, 39.40 mmol), Ac_2_O (25.58 g, 251.00 mmol), AcOH (12.04 g, 201.00 mmol) was refluxed for 3 h. Compound **16** (20.00 g, 71.6 mmol) was added to the resulting solution and the mixture was refluxed for 2 h, then toluene (0.12 L) was added. After cooling to room temperature, the resulting precipitates were collected by filtration to give an off-white solid (25.00 g, 86%). ^1^H-NMR (400 MHz, CDCl_3_) δ 9.21 (s, 1H), 8.27–8.21 (m, 1H), 5.05–4.93 (m, 1H), 3.39–3.30 (m, 1H), 3.17–3.06 (m, 1H), 2.38–2.31 (m, 2H), 2.07 (s, 3H), 2.01 (s, 3H), 1.64 (d, *J* = 6.8 Hz, 3H). ESI-MS (*m*/*z*): 405.9 [M + H]^+^.

OBP-4: A mixture of compound **17** (14.00 g, 34.40 mmol), compound **8** (15.20 g, 44.70 mmol) and TEA (10.40 g, 0.103 mol) in DMF (0.25 L) was stirred at 50 °C for 12 h. After cooling to room temperature, the mixture was poured into water (2.50 L), the resulting precipitates were collected by filtration and dried to give a yellow solid (21.60 g, 86%), the yellow solid was added to a mixture of 1.5 N aqueous LiOH (0.08 L) and ACN (0.04 L). The mixture was stirred at room temperature for about 2 h and then concentrated HCl was added until PH is around 2, the resulting precipitates were collected by filtration, the obtained solid was suspended in 50% (*v*/*v*) aqueous ethanol and refluxed for 6 h, after cooling to room temperature, the mixture was filtered to give a white solid, which was recrystallized with ACN and EtOH to give a white solid (10.25 g, 50%), m.p., 225–226 °C. ^1^H-NMR (400 MHz, DMSO-*d*_6_) δ 15.32 (s, 1H), 8.96 (s, 1H), 7.87 (d, *J* = 12.5 Hz, 1H), 7.59 (d, *J* = 13.8 Hz, 1H), 7.27–7.21 (m, 2H), 5.23 (t, *J* = 5.5 Hz, 1H), 4.88 (br s, 1H), 4.81 (s, 1H), 4.69 (br s, 1H), 4.05 (t, *J* = 8.9 Hz, 1H), 3.91 (s, 2H), 3.82–3.78 (m, 1H), 3.74–3.66 (m, 2H), 3.58–3.53 (m, 1H), 3.29 (s, 1H), 3.16–2.94 (m, 4H), 2.16–1.94 (m, 3H), 1.80 (d, *J* = 10.3 Hz, 1H), 1.68 (t, *J* = 13.2 Hz, 2H), 1.42 (d, *J* = 6.5 Hz, 3H). ^13^C-NMR (151 MHz, DMSO-*d*_6_) δ176.02 (d, *J* = 3.02 Hz), 166.26, 163.59, 154.87, 152.29 (d, *J* = 246.13 Hz), 151.91 (d, *J* = 243.11 Hz), 145.97 (d, *J* = 10.57 Hz), 143.11 (d, *J* = 10.57 Hz), 136.33, 132.75 (d, *J* = 9.06 Hz), 117.26 (d, *J* = 7.55 Hz), 116.32, 114.23, 112.43 (d, *J* = 22.65 Hz), 107.06 (d, *J* = 24.16 Hz), 102.72, 101.96, 77.35, 73.57, 72.72, 67.98, 62.08, 46.62, 45.91, 45.76, 33.52, 33.46, 20.77. ESI-MS (*m*/*z*) [M + H]^+^ calcd. for C_30_H_31_F_2_N_3_O_8_: 600.2152, found: 600.2159.

#### 3.2.3. Preparation of Hybrid OBP-5

Compound **18**: to a mixture of ethyl 6,7-difluoro-1-methyl-4-oxo-4H-(1,3)-thiazeto (3,2-a) quinoline-3-carboxylate (3.78 g, 12.10 mmol) [31], compound **8** (4.00 g, 11.80 mmol), TEA (3.57 g, 35.30 mmol) and DMSO (0.06 L) was stirred at 100 °C overnight. The reaction was quenched with water (0.12 L), the mixture was filtered to give compound **18** (6.15 g, 83%) as a white solid. ^1^H-NMR (400 MHz, DMSO-*d*_6_) δ 7.66–7.70 (d, *J* = 16 Hz,1H), 7.57–7.61 (d, *J* = 16 Hz,1H), 6.84–6.85 (d, *J* = 4 Hz, 1H), 6.17–6.21 (q, *J* = 12, 4Hz, 1H), 5.23–5.25 (t, *J* = 8 Hz, 1H), 4.86 (s, 1H), 4.68–4.70 (dd, *J* = 8, 4 Hz, 1H), 4.18 (q, *J* = 16 Hz, 2H), 4.06 (t, *J* = 12 Hz, 1H), 3.90 (s, 2H), 3.83 (s, 1H), 3.67 (m,1H), 3.55–3.59 (m, 1H), 3.43–3.46 (m, 2H), 3.17–3.25 (dd, *J* = 24, 12 Hz, 2H), 2.06 (d, *J* = 4 Hz, 3H), 1.88–1.93(t, *J* = 12 Hz,2H), 1.69–1.72 (d, *J* = 12 Hz, 2H), 1.24–1.26 (t, *J* = 8 Hz, 3H). MS (ESI): mass calcd.. for C_30_H_31_F_2_N_3_O_8_S 631.18, *m*/*z* found: 632.1 [M + H]^+^.

OBP-5: compound **18** (1.58 g, 2.50 mmol, 1eq) was dissolved in a mixture of THF/MeOH (0.02 L mL/0.02 L), and then lithium hydroxide (2.00M, 0.02 L) was slowly added and stirred at room temperature for 2 h. The reaction mixture was filtered to give the crude of OBP-5 as a solid after adjusting its pH (<6) with 50% H_2_SO_4_. The crude was separated on a silica gel column (DCM: MeOH=1:1) to obtain light white solid OBP-5 (0.38 g, 25%), m.p., 250–251 °C. ^1^H-NMR (400 MHz, DMSO-*d*_6_) δ 14.70 (s, 1H), 7.79 (d, *J* = 13.6 Hz, 1H), 7.58 (d, *J* = 14.4 Hz, 1H), 7.27–7.17 (m, 2H), 6.96 (d, *J* = 6.4 Hz, 1H), 6.40 (s, 1H), 5.23 (t, *J* = 5.6 Hz, 1H), 4.90 (s, 1H), 4.68 (dd, *J* = 8.8, 5.4 Hz, 1H), 4.04 (t, *J* = 8.8 Hz, 1H), 3.90 (s, 2H), 3.82–3.75 (m, 1H), 3.71–3.62 (m, 1H), 3.57–3.50 (m, 3H), 3.29–3.23 (m, 2H), 2.11 (d, *J* = 5.6 Hz, 3H), 1.90 (t, *J* = 10.8 Hz, 2H), 1.71 (d, *J* = 12.8 Hz, 2H).^13^C-NMR (151 MHz, DMSO-*d*_6_) δ176.98 (d, *J* = 3.02 Hz), 166.55, 154.88, 151.99 (d, *J* = 242.11 Hz), 147.81, 143.31 (d, *J* = 12.08 Hz), 143.22 (d, *J* = 10.57 Hz), 133.45(d, *J* = 199.32 Hz), 132.73, 122.32, 116.56 (d, *J* = 6.04 Hz), 114.25 (d, *J* = 3.02 Hz), 109.40 (d, *J* = 22.65 Hz), 107.06 (d, *J* = 24.16 Hz), 106.94, 73.57, 68.19, 62.09, 57.54, 46.87, 46.62, 34.86, 34.33, 25.35, 20.03, 18.99. ESI-MS (*m*/*z*) [M + H]^+^ calcd. for C_28_H_27_F_2_N_3_O_8_S: 604.1560, found: 604.1565.

#### 3.2.4. Preparation of Hybrids OBP-6 and OBP-7

Compound **19**: to a mixture of *N*-benzylmaleimide (75.00 g, 401.07 mmol, 1 eq) [32] and BrCH_2_NO_2_ (45.00 g, 320.09 mmol, 0.8 eq), K_2_CO_3_ (44.00 g, 320.09 mmol, 0.8 eq) and ACN (0.40 L) were slowly added and then stirred at 25 °C for 48 h. The reaction mixture was extracted with DCM (0.30 L*3) and brine (0.30 L). The combined organic layers were dried with anhydrous Na_2_SO_4_ and concentrated to give compound **19** (17.00 g, 17%) as a yellow oil. ^1^H-NMR (400 MHz, CDCl_3_) δ 7.36–7.27 (m, 5H), 4.54 (s, 2H), 4.50–4.46 (m, 1H), 3.35 (s, 2H). ESI-MS (*m*/*z*): 246.9 [M + H]^+^.

Compound **20**: to a mixed solution of compound **19** (16.30 g, 66.26 mmol, 1 eq) and NaBH4 (6.29 g, 165.65 mmol, 2.5 eq) in THF (0.40 L), BF3·Et_2_O (8.36 mL, 66.26 mmol, 1 eq) was added dropwise at 0 °C and maintained for 1 h. The mixture was heated to 40 °C and kept for 4 h. After cooling, the reaction mixture was treated with EtOH (0.40 L) under reflux for 4 h. The mixture was extracted with DCM (0.30 L*3) and brine (0.30 L). The combined organic layers were dried with anhydrous Na_2_SO_4_. After evaporation, compound **20** (9.00 g, 60%) was obtained by flash chromatography as a colorless oil. ^1^H-NMR (400 MHz, CDCl_3_) δ 7.35–7.20 (m, 5H), 4.63 (s, 1H), 3.59 (s, 2H), 3.14 (d, *J* = 9.2 Hz, 2H), 2.51 (d, *J* = 8.0 Hz, 4H). ESI-MS (*m*/*z*): 218.9 [M + H]^+^.

Compound **21**: Zn (39.20 g, 599.48 mmol, 20 eq) was slowly added to a solution of compound **20** (6.54 g, 300.00 mmol, 1 eq) and 1N HCl (0.30 L, 0.30 mol, 10 eq) in i-PrOH (0.60 L) within 1 h, and kept overnight. Excess hydrochioric acid was neutralized using NaHCO_3_ until pH > 7. The reaction was extracted with DCM (0.30 L*3) and brine (0.30 L). The combined organic layers were dried with anhydrous Na_2_SO_4_. After evaporation, compound **21** (5.56 g crude) was obtained by flash chromatography as a yellow oil. ^1^H-NMR (400 MHz, CDCl_3_) δ 7.31–7.18 (m, 5H), 3.55 (s, 2H), 2.95 (d, *J* = 8.8 Hz, 2H), 2.66 (s, 1H), 2.38 (d, *J* = 8.4 Hz, 2H), 1.34 (s, 2H).

Compound **22**: DIPEA (7.63 g, 0.06 mol, 2 eq) was added dropwise to a solution of compound **21** (5.56 g, 0.03 mol, 1 eq) and 2,4-difluoronitrobenzene in ACN (0.10 L) and stirred at 50 °C for 48 h. After cooling to room temperature, the reaction mixture was extracted with DCM (0.30 L*3) and brine (0.30 L). The combined organic layers were dried with anhydrous Na_2_SO_4_. After evaporation, compound **22** (9.10 g, 93%) was obtained by flash chromatography as a yellow oil. ^1^H-NMR (400 MHz, CDCl_3_) δ 8.00 (d, *J* = 8.8 Hz, 1H), 7.86 (dd, *J* = 11.6, 2.4 Hz, 1H), 7.38–7.28 (m, 5H), 6.93 (t, *J* = 8.8 Hz, 1H), 4.85 (s, 1H), 3.79–3.60 (m, 2H), 3.25 (s, 2H), 3.03 (s, 1H), 2.61 (s, 2H), 1.26 (s, 2H).

Compound **23**: to a mixture of compound **22** (9.10 g, 0.03 mol, 1 eq), (Boc)_2_O (9.11 g, 0.04 mol, 1.5 eq) and TEA (5.60 g, 56.00 mmol, 2 eq) in DCM (0.20 L), DMAP (340.00 mg, 3.00 mmol, 0.1 eq) was added at 0 °C and stirred for 2 h. The reaction mixture was extracted with DCM (0.30 L*3) and brine (0.30 L). The combined organic layers were dried with anhydrous Na_2_SO_4_ and then concentrated to give a yellow oil. NaBH_4_ (3.20 g, 80.00 mmol, 3 eq) and NiCl_2_·6H_2_O (20.00 g, 80.00 mmol, 3eq) were separately added to the oil in THF/MeOH (0.05 L/0.05 L) at 0 °C and stirred for 2 h. The reaction mixture was extracted with EA (0.30 L*3) and brine (0.30 L). The combined organic layers were dried with anhydrous Na_2_SO_4_. After evaporation, compound **23** (10.00 g, 91%) was obtained by flash chromatography as a yellow oil. ^1^H-NMR (400 MHz, CDCl_3_) δ 7.31–7.22 (m, 5H), 6.87 (t, *J* = 8.2 Hz, 1H), 6.38 (d, *J* = 9.6 Hz, 2H), 3.72 (s, 2H), 3.55 (s, 2H), 3.25 (s, 1H), 3.03 (d, *J* = 8.8 Hz, 2H), 2.37 (d, *J* = 7.6 Hz, 2H), 1.53 (s, 2H), 1.41 (s, 9H).

Compound **24**: the saturated NaHCO_3_ solution was added to compound **23** (10.00 g, 30.00 mmol, 1 eq) in THF (0.50 L). CBzCl (5.16 g, 36.00 mmol, 1.2 eq) was slowly added to the mixed solution at 0 °C and stirred overnight. The reaction mixture was extracted with EA (0.30 L*3) and brine (0.30 L). The combined organic layers were dried with anhydrous Na_2_SO_4_. After evaporation, compound **24** (10.00 g, 75%) was obtained by flash chromatography as a yellow oil. ^1^H-NMR (400 MHz, CDCl_3_) δ 7.41–7.38 (m, 3H), 7.26 (s, 10H), 5.20 (s, 2H), 3.54 (s, 2H), 3.28 (s, 1H), 3.02 (d, *J* = 8.8 Hz, 2H), 2.35 (d, *J* = 8.8 Hz, 2H), 2.02 (s, 1H), 1.56 (s, 9H), 1.34 (s, 2H).

Compound **25**: the suspension of compound **24** (10.00 g, 18.83 mmol, 1 eq) in 0.30 L of THF was cooled to −78 °C. n-BuLi (2.50 M, 23.00 mL, 56.50 mmol, 3 eq) was added dropwisely to the mixed solution within 30 min. After stirring at −78 °C for 30 min, (R)-oxiran-2-ylmethyl butyrate in THF (0.30 L) was added dropwisely within 30 min and stirred at −78 °C for 30 min. After being restored to room temperature, the reaction solution was continuously stirred for 12 h. The resulting mixture was extracted with EA (0.30 L*3) and brine (0.30 L). The combined organic layers were dried with anhydrous MgSO_4_. After evaporation, compound **25** (3.50 g, 37%) was obtained by flash chromatography as a yellow oil. ^1^H-NMR (400 MHz, CDCl_3_) δ 7.51 (d, *J* = 11.2 Hz, 1H), 7.36–7.27 (m, 5H), 7.16 (s, 2H), 4.75 (s, 1H), 4.10–3.98 (m, 3H), 3.76 (d, *J* = 12.4 Hz, 1H), 3.64 (s, 2H), 3.34 (s, 1H), 3.09 (s, 2H), 2.50 (s, 2H), 1.42 (s, 9H), 1.34 (s, 2H).

Compound **26**: Pd/C (0.35 g, 10%) was carefully added to a solution of compound **25** (3.50 g, 7.04 mmol, 1 eq) in MeOH (0.05 L). The reaction was proceeded with H_2_ at room temperature for 12 h. The liquid was filtered and concentrated to afford compound **26** (2.50 g crude, 87%) as a yellow oil. ESI-MS (*m*/*z*): 408.0 [M + H]^+^.

Compound **27**: to a mixture of compound **26** (1.20 g, 2.95 mmol, 1 eq) and 9,10-difluoro-3- methyl-7-oxo-2,3-dihydro-7H-[1,3,4] oxadiazino[6,5,4-ij]quinoline-6-carboxylic acid (0.831 g, 2.95 mmol, 1 eq) [33] in DMSO (0.02 L), TEA (1.50 g, 14.74 mmol, 5 eq) was added and stirred at 100 °C overnight. Excess TEA was neutralized using 50% H_2_SO_4_ until pH < 6. The filtrate was concentrated and the residue was purified by silica gel column to afford compound **27** (0.35 g, 18%) as a yellow oil. ^1^H-NMR (400 MHz, DMSO-*d*_6_) δ 15.13 (s, 1H), 8.71 (s, 1H), 7.58 (t, *J* = 13.6 Hz, 2H), 7.36–7.30 (m, 2H), 5.26 (s, 2H), 4.71 (s, 1H), 4.10 (t, *J* = 8.8 Hz, 1H), 3.91–3.81 (m, 2H), 3.71–3.60 (m, 3H), 3.57 (d, *J* = 3.6 Hz, 1H), 3.54 (d, *J* = 3.6 Hz, 1H), 3.50 (s, 2H), 2.99 (s, 3H), 1.74 (s, 2H), 1.38 (s, 9H).

Compound **28**: to a mixture of compound **26** (1.30 g, 3.19 mmol, 1 eq) and 1-cyclopropyl-6,7- difluoro-4-oxo-1,4-dihydroquinoline-3-carboxylic acid (0.846 g, 3.19 mmol, 1 eq) [34] in DMSO (0.02 L), TEA (1.63 g, 15.97 mmol, 5 eq) was added and stirred at 100 °C overnight. After cooling to room temperature, excess TEA was neutralized using 50% H_2_SO_4_ until pH < 6. After concentration of the solution, the residue was purified by silica gel column to afford compound **28** (0.35 g, 17%) as a white solid. ^1^H-NMR (400 MHz, CDCl_3_) δ 8.58 (s, 1H), 7.80 (d, *J* = 14.4 Hz, 1H), 7.61 (d, *J* = 12.4 Hz, 1H), 7.40 (t, *J* = 8.6 Hz, 1H), 7.34 (d, *J* = 10.4 Hz, 1H), 7.07 (d, *J* = 7.2 Hz, 1H), 5.26 (d, *J* = 5.2 Hz, 1H), 4.77–4.70 (m, 1H), 4.10 (t, *J* = 9.0 Hz, 1H), 3.8–3.80 (m, 3H), 3.75–3.64 (m, 4H), 3.58–3.50 (m, 3H), 1.93 (s, 2H), 1.39 (s, 9H), 1.23 (s, 2H), 1.12 (s, 2H).

OBP-6: to a solution of compound **27** (0.35 g, 0.52 mmol, 1 eq) in THF (0.03 L), 6N HCl (0.03 L) was added dropwise at 0 °C and then stirred for 48 h at room temperature. The saturated NaHCO_3_ solution was added in ice bath to adjust pH (>7). The mixture was extracted with EA (0.30 L*3) and brine (0.30 L). The combined organic layers were dried with anhydrous Na_2_SO_4_. After evaporation, the residue was purified by silica gel column to afford hybrid OBP-6 as a yellow solid (0.28 g crude, 94%), m.p., 188–189 °C. ^1^H-NMR (400 MHz, DMSO-*d*_6_) δ 8.67 (s, 1H), 7.51 (d, *J* = 13.6 Hz, 1H), 7.39 (d, *J* = 13.6 Hz, 1H), 7.13 (d, *J* = 8.8 Hz, 1H), 6.91 (t, *J* = 9.2 Hz, 1H), 5.92 (s, 1H), 5.21 (s, 3H), 4.64 (s, 1H), 3.98 (d, *J* = 8.8 Hz, 2H), 3.79–3.70 (m, 3H), 3.65 (d, *J* = 11.6 Hz, 2H), 3.54 (d, *J* = 11.2 Hz, 2H), 2.99 (s, 3H), 1.78 (s, 2H). ^13^C-NMR (151 MHz, DMSO-*d*_6_) δ 176.28 (d, *J* = 3.02 Hz), 166.03, 154.97, 154.37 (d, *J* = 206.87 Hz), 150.55 (d, *J* = 241.60 Hz), 145.42, 136.44 (d, *J* = 9.06 Hz), 133.49 (d, *J* = 12.08 Hz), 128.65 (d, *J* = 4.53 Hz), 124.37, 115.51, 113.13 (d, *J* = 4.53 Hz), 106.52 (d, *J* = 24.16 Hz), 104.35 (d, *J* = 24.16Hz), 82.48, 73.45, 62.11, 52.11, 46.85, 43.16, 43.01, 33.84, 31.12, 24.35, 21.58. ESI-MS (*m*/*z*) [M + H]^+^ calcd. for C_2__7_H_25_F_2_N_5_O_7_: 570.1795, found: 570.1804.

OBP-7: to a solution of compound **28** (0.35 g, 0.54 mmol, 1 eq) in THF (0.01 L), EA/HCl (0.03 L) was added dropwise and then stirred for 48 h at room temperature. The reaction mixture was filtered and the yellow solid OBP-7 was obtained (0.25 g crude, 84%) by recrystallization in PE and EA (1:1), m.p., 268–270 °C. ^1^H-NMR (400 MHz, DMSO-*d*_6_) δ 8.58 (s, 1H), 7.80 (d, *J* = 14.4 Hz, 1H), 7.41 (d, *J* = 14.4 Hz, 1H), 7.13 (d, *J* = 5.6 Hz, 2H), 6.99 (t, *J* = 9.2 Hz, 1H), 4.69–4.61 (m, 1H), 4.42–4.25 (m, 1H), 4.03–4.01 (m, 3H), 3.77 (d, *J* = 7.6 Hz, 4H), 3.69–3.61 (m, 2H), 3.61–3.48 (m, 2H), 1.96 (s, 2H), 1.33 (d, *J* = 6.8 Hz, 2H), 1.16 (s, 2H). ^13^C-NMR (151 MHz, DMSO-*d*_6_) δ 176.21 (d, *J* = 3.02 Hz), 166.54, 154.97, 150.53 (d, *J* = 238.58 Hz), 150.20 (d, *J* = 247.64 Hz), 148.18, 141.65 (d, *J* = 12.08 Hz), 139.96, 133.28 (d, *J* = 12.08 Hz), 128.79 (d, *J* = 9.06 Hz), 115.21, 115.18, 113.37 (d, *J* = 4.53 Hz), 111.12 (d, *J* = 24.16 Hz), 109.99, 106.62, 106.50 (d, *J* = 24.16 Hz), 102.02, 73.46, 62.12, 52.09, 46.83, 36.49, 36.09, 25.62, 8.21. ESI-MS (*m*/*z*) [M + H]^+^ calcd. for C_28_H_26_F_2_N_4_O_6_: 553.1893, found: 553.1901.

### 3.3. Antibacterial Activities

#### 3.3.1. Bacterial Strains

6 standard strains and 3 clinical isolates were used to the preliminary evaluation of in vitro antibacterial activities of oxazolidinone-fluoroquinolone hybrids. For the further evaluation of hybrids OBP-4 and OBP-5, 263 clinical isolates including *S. aureus* (46), *S. haemolyticus*(32), *S. epidermidis* (10), *E. faecalis* (72), *E. faecium* (47), *S. pneumoniae* (13), *H. parasuis* (34), *S. suis* (9), and 8 resistant strains including MRSA, MRSE, CIP-resistant *S. aureus and S. haemolyticus*, VAN and quinolone-resistant *S. pneumoniae*, LZD-resistant *E. faecalis*, LZD/VAN and quinolone-resistant *E. faecium* were collected and tested.

#### 3.3.2. Susceptibility Testing

Susceptibility testing of oxazolidinone-fluoroquinolone hybrids against various bacteria was performed by the broth microdilution method as outlined by the Clinical and Laboratory Standards Institute (CLSI) [35]. Three types of media, including cation-adjusted Mueller Hinton broth, Haemophilus Test Medium and cation-adjusted Mueller Hinton broth that contained 3%–5% lysed horse blood, were used to culture the corresponding bacteria. All compounds including comparator drugs were dissolved in an appropriate solvent at a concentration of 2.048 mg/mL and then serially diluted two-fold to provide the required concentrations. A final inoculum of 5 × 10^5^ CFU/mL was used and plates were read after incubation for 16–24 h at 35 °C. The MIC was defined as the lowest concentration of the antibacterial at which visible growth was completely inhibited. Standard quality control strains (ATCC) were included throughout. Each determination was repeated in triplicate.

### 3.4. Modes of Action

#### 3.4.1. DNA Gyrase Supercoiling Assay

The *E. coli* DNA Gyrase Supercoiling Assay Kit (Inspiralis Co., Ltd.) was used to investigate the inhibition activities of hybrids OBP-4 and OBP-5 against DNA gyrase. DNA gyrase is prepared from *E. coli*. One unit of DNA gyrase can convert 0.5 μg of relaxed pBR322 to the supercoiled forms in 30 min. The final reaction system (30 μL) consisted of 0.5 μg relaxed pBR322 and 1 unit DNA gyrase with or without compounds in assay buffer containing 35 mM Tris·HCl (pH 7.5), 24 mM KCl, 4 mM MgCl_2_, 2 mM DTT, 1.8 mM spermidine, 1 mM ATP, 6.5% (*w*/*v*) glycerol and 0.1 mg/mL albumin, and then was incubated at 37 °C for 30 min. The reaction was terminated by adding 30 μL of STEB and 30 μL of chloroform/isoamyl alcohol (*v*:*v*, 24:1), and then the products were resolved by gel electrophoresis on 1% (*w*/*v*) agarose gel at 80 V for 2 h in the absence of ethidium bromide. The gels were stained with ethidium bromide (1 μg/mL in distilled water) for 15 min, and destained (5–10 min) in distilled water. DNA bands were visualized with UV light in Bio-Rad^®^ Chemidoc xrs and analyzed by using Quantity One software (Version 4.6, Bio-Rad). IC_50_ is defined as the concentration of compound that inhibits the enzyme activity by 50%. CIP was used as a positive control.

#### 3.4.2. Topo IV Relaxation Assay

The *E. coli* Topo IV Relaxation Assay Kit (Inspiralis Co., Ltd.) was used to evaluate the effects of hybrids OBP-4 and OBP-5 against topo IV. Topo IV was prepared by the overexpressing *E. coli*. One unit of topo IV will relax 0.4 μg of supercoiled pBR322. In the assay, the final reaction mixtures (30 μL) consisted of 0.4 μg supercoiled pBR322 and one unit DNA Topo IV with or without compounds in assay buffer with 40 mM Hepes-KOH (pH 7.6), 100 mM potassium glutamate, 10 mM magnesium acetate, 10 mM DTT, 1 mM ATP and 50 μg/mL albumin, and then was incubated at 37 °C for 30 min. The reaction was terminated by adding 30 μL of STEB and 30 μL of chloroform/isoamyl alcohol (*v*:*v*, 24:1) after incubation for 30 min. Then, the reaction products were processed as described in a DNA gyrase supercoiling assay.

#### 3.4.3. In Vitro Transcription/Translation Assay

The influence of hybrids OBP-4 and OBP-5 against protein synthesis was confirmed by the S30 T7 High-Yield Protein Expression System (Promega Co., Ltd.). The S30 T7 High-Yield Protein Expression System is an *E. coli* extract-based cell-free protein expression system, which can effectively transcribe and translate DNA fragments with a T7 promoter. The reaction system containing 1 μg plasmid DNA, 20 μL S30 Premix Plus and 18 μL T7 S30 Extract with or without compounds was incubated for 1 h at 37 °C with a rotational speed of 250 rpm, and terminated by cooling in ice for 5 min in a 96-well white multi-well plate. Then, 1.25 μL of the translation reaction mixture was added to 48.75 μL of Renilla Luciferase Assay Lysis Buffer, and mixed with 50 μL of Renilla Luciferase Assay Reagent (cat no. E2810; Promega). Renilla luciferase activity was measured on EnSpire^®^ Multimode Plate Reader (PerkinElmer, Waltham, MA, USA). IC_50_ is defined as the concentration of compound that inhibits the protein synthesis by 50%. LZD was used as a positive control.

#### 3.4.4. Molecular Docking Stusy

The molecular docking of hybrids OBP-4 and OBP-5 was carried out with the Discovery Studio 4.5 software. The crystal structure of the large ribosomal subunit (50S subunit) of *S. aureus* (PDB: 4WFA) [36] was selected as the template to perform the docking study. Prior to docking, the structures of ligands were drawn and energy was minimized. During the process of receptor preparation, water molecules were removed and hydrogen atoms were added [37]. Hit compounds were docked into the active site of the receptor with an acceptable target flexibility and the docking score was calculated. The docking complexes with the highest score were kept for further analyses. The presentation of ligand-receptor interactions was depicted by PyMol 1.5.03 [38].

### 3.5. Acute Toxicity Test

Female Kunming mice (SPF grade, 18–22 g) were obtained from laboratory animal center of Lanzhou Veterinary Research Institute (Lanzhou, China). Mice were maintained under the standard conditions of 12 h light/dark cycle, room temperate (22 ± 3 °C), 50–60% relative humidity. They were fed normally with an unlimited supply of feed and water for seven days and deprived of food but not water for 4 h prior to the experiment. The toxicity test was supported by the Animal Ethics Committee of Lanzhou Institute of Husbandry and Pharmaceutical Sciences.

The acute toxicity of hybrids OBP-4 and OBP-5 was conducted by the limit test described in the OECD guideline 425 [39]. The mice were divided randomly into two groups, five animals in each group. One group of mice received a single oral dose of hybrids OBP-4 or OBP-5 at 2000 mg/kg·bw, and the other group of mice was given the equal volume of 0.5% CMC-Na solution (0.1 mL/10 g of body weight). Food was restored until 4 h after administration. The observation of each animal occurred according to the OECD guideline 425 [27], meaning continuously during the first 30 min, periodically during the next 24 h, and then daily thereafter until 14 d after dosing. The weight changes and mortality were calculated and recorded. The mice that survived were humanely killed at the end of the experiment.

## 4. Conclusions

A series of new oxazolidinone-fluoroquinolone hybrids incorporating the structural elements of oxazolidinone and fluoroquinolone with a linker were synthesized and evaluated for their in vitro antibacterial activities against various Gram-positive and Gram-negative bacteria. The antibacterial activities of most hybrids against Gram-positive bacteria were significantly higher than that of LZD, and hybrids OBP-4 and OBP-5 were the most active two hybrids. The mode of action for hybrids OBP-4 and OBP-5 was more directed to protein synthesis inhibition instead of DNA synthesis inhibition, showing a good consistency with the antibacterial profile. Meanwhile, a good safety profile was also demonstrated for hybrids OBP-4 and OBP-5 with LD_50_ values greater than 2000 mg/kg. These combined data suggested that hybrids OBP-4 and OBP-5 may be promising therapeutic options for severe nosocomial infections caused by Gram-positive bacteria.

## Figures and Tables

**Table 1:** In vitro antibacterial activities of oxazolidinone-fluoroquinolone hybrids.

Organism (No.)	MIC (µg/mL)									
	OBP-1	OBP-2	OBP-3	OBP-4	OBP-5	OBP-6	OBP-7	CZD	LZD	MXF
*S. pneumoniae* (ATCC49619)	0.25	0.25	0.5	0.031	0.062	0.5	2	0.062	1	0.062
*S. agalactiae*^R^ (13B317)	0.5	0.25	0.5	0.031	0.031	2	4	0.125	1	4
*E. faecalis* (ATCC29212)	1	1	1	0.25	1	4	1	0.5	4	0.125
VRE (13B330)	1	1	2	0.25	0.5	64	128	0.25	2	64
MRSA (13B262)	2	1	2	0.5	0.5	64	128	0.5	4	8
*S. aureus* (ATCC29213)	1	0.25	2	0.016	0.25	0.062	0.25	0.5	4	0.031
*H. influenzae* (ATCC49247)	1	0.5	2	0.25	0.25	0.016	0.031	1	8	0.008
*P. Aeruginosa* (ATCC27853)	64	64	128	64	64	16	64	64	128	4
*E. coli* (ATCC25922)	4	4	64	4	4	1	0.5	8	128	0.016

*S. agalactiae*^R^ (13B317), levofloxacin-resistant *S. agalactiae.*

**Table 2:** In vitro activities of OBP-4 and OBP-5 against clinical isolates.

Organism (No. Tested)	Agents	MIC (µg/mL)			CLSI ^a^ %S/%R
		MIC_50_	MIC_90_	Range	
*S. aureus* (46)	OBP-4	≤0.0625	0.125	≤0.0625–0.25	-/-
	OBP-5	≤0.0625	≤0.0625	≤0.0625–0.25	-/-
	ENR	2	4	≤0.0625–8	
	CIP	16	32	≤0.0625–32	37.9/62.1
	GAFX	1	2	≤0.0625–2	45.5/42.4
	NDFX	0.5	0.5	≤0.0625–2	
	LZD	2	2	0.5–2	100.0/-
	VAN	0.5	0.5	0.25–0.5	100.0/-
*S. haemolyticus* (32)	OBP-4	0.125	0.125	≤0.0625–0.5	-/-
	OBP-5	≤0.0625	≤0.0625	≤0.0625–0.125	-/-
	ENR	≤0.0625	0.5	≤0.0625–8	
	CIP	0.25	1	0.125–16	96.2/3.8
	GAFX	0.125	0.25	≤0.0625–2	96.2/3.8
	NDFX	≤0.0625	0.5	≤0.0625–4	
	LZD	0.5	2	0.125–8	96.2/3.8
	VAN	0.5	2	≤0.0625–4	96.2/-
*S. epidermidis* (10)	OBP-4	0.125	0.25	≤0.0625–0.25	-/-
	OBP-5	≤0.0625	0.125	≤0.0625–0.125	-/-
	ENR	2	4	0.125–4	
	CIP	8	16	0.25–16	20.0/60.0
	GAFX	1	2	0.125–2	30.0/50.0
	NDFX	1	2	≤0.0625–2	
	LZD	1	1	0.5–1	100.0/-
	VAN	1	2	1–2	100.0/-
*S. suis* (9)	OBP-4	≤0.0625	0.25	≤0.0625–2	-/-
	OBP-5	≤0.0625	0.125	≤0.0625–2	-/-
	ENR	0.25	4	≤0.0625–8	
	CIP	0.25	8	0.125–16	
	GAFX	≤0.0625	4	≤0.0625–4	
	NDFX	≤0.0625	2	≤0.0625–4	
	LZD	0.5	4	0.5–4	
	VAN	0.25	2	0.125–2	
*S. pneumoniae* (13)	OBP-4	0.25	0.25	≤0.0625–0.5	-/-
	OBP-5	≤0.0625	0.25	≤0.0625–0.25	-/-
	ENR	32	>32	2–>32	
	CIP	16	>32	4–>32	-/100.0
	GAFX	4	32	4–32	7.7/92.3
	NDFX	16	>32	8–>32	
	LZD	0.5	0.5	0.5–4	76.9/-
	VAN	1	32	0.5–>32	53.8/-
*E. faecalis* (72)	OBP-4	0.25	0.25	≤0.0625–1	-/-
	OBP-5	0.125	0.125	≤0.0625–0.5	-/-
	ENR	0.5	0.5	≤0.0625–2	
	CIP	0.5	1	0.125–2	97.3/-
	GAFX	0.25	0.5	0.125–2	94.6/-
	NDFX	0.25	1	0.125–2	
	LZD	2	2	0.5–8	97.3/2.7
*E. faecium* (47)	OBP-4	0.5	0.25	0.25–2	-/-
	OBP-5	0.25	0.25	≤0.0625–1	-/-
	ENR	2	32	0.5–>32	
	CIP	2	32	0.25–>32	51.7/37.9
	GAFX	1	8	0.25–8	86.2/13.8
	NDFX	2	8	0.5–16	
	LZD	2	4	0.5–16	82.8/13.8
	VAN	2	4	0.5–4	100.0/-
*H. parasuis* (34)	OBP-4	1	4	≤0.0625–4	-/-
	OBP-5	0.5	2	≤0.0625–4	-/-
	ENR	≤0.0625	1	≤0.0625–1	
	CIP	≤0.0625	1	≤0.0625–1	
	GAFX	≤0.0625	1	≤0.0625–1	
	NDFX	≤0.0625	1	≤0.0625–1	
	LZD	16	>32	8–>32	
	VAN	8	>32	2–>32	

^a^ Criteria as published by CLSI. S, susceptible; R, resistant. Empty cells indicate that no breakpoints have been defined.

**Table 3:** MIC frequency distributions for tests of OBP-4 and OBP-5 against clinical isolates.

Organism (No. Tested)	Agents	No. of Strains (Cumulative %) Inhibited at Each MIC (µg/mL)						
		≤0.0625	0.125	0.25	0.5	1	2	4
*S. aureus* (46)	OBP-4	26(56.5)	17(93.5)	3(100.0)				
	OBP-5	42(91.3)	3(97.8)	1(100.0)				
*S. haemolyticus* (32)	OBP-4	14(43.8)	16(93.8)		2(100.0)			
	OBP-5	30(93.4)	2(100.0)					
*S. epidermidis* (10)	OBP-4	2(20.0)	4(60.0)	4(100.0)				
	OBP-5	8(80.0)	2(20.0)					
*S. suis* (9)	OBP-4	5(55.6)	2(77.8)	1(88.9)			1(100)	
	OBP-5	5(55.6)	3(88.9)				1(100)	
*S. pneumoniae* (13)	OBP-4	2(15.4)	3(38.5)	7(92.3)	1(100.0)			
	OBP-5	7(53.8)	4(84.6)	2(100.0)				
*E. faecalis* (72)	OBP-4	9(12.5)	17(36.1)	43(95.8)	2(98.6)	1(100.0)		
	OBP-5	17(23.6)	49(91.7)	5(98.6)	1(100.0)			
*E. faecium* (47)	OBP-4			8(17.2)	37(95.7)	1(97.9)	1(100.0)	
	OBP-5	3(6.4)	7(21.3)	33(91.5)	3(97.9)	1(100.0)		
*H. parasuis* (34)	OBP-4	4(11.8)		3(20.6)	5(35.3)	9(61.8)	6(79.4)	7(100.0)
	OBP-5	5(14.7)	1(17.6)	4(29.4)	6(47.1)	7(67.6)	10(97.1)	1(100.0)

**Table 4:** In vitro activities of OBP-4 and OBP-5 against drug-resistant strains.

Organism	MIC (µg/mL)							
	OBP-4	OBP-5	ENR	CIP	GAFX	NDFX	LZD	VAN
MRSA	0.25	0.125	0.25	0.125	0.125	≤0.0625	4	1
MRSE	≤0.0625	≤0.0625	≤0.0625	0.125	≤0.0625	0.125	0.25	1
*S. aureus* ^a^	0.125	≤0.0625	2	32	2	0.5	2	0.25
*S. haemolyticus* ^a^	0.5	≤0.0625	8	16	2	4	8	2
*S. pneumoniae* ^b^	0.25	≤0.0625	>32	>32	>32	>32	0.5	>32
*E. faecaliss* ^c^	1	0.25	1	1	0.5	1	8	2
*E. faecium* ^d^	0.5	0.125	>32	>32	8	16	16	4
*E. faecium* ^b^	0.5	0.25	>32	>32	16	32	2	16

^a^ CIP-resistant; ^b^ VAN and quinolone-resistant; ^c^ LZD-resistant; ^d^ LZD and quinolone-resistant.

**Table 5:** Activity of OBP-4 and OBP-5 against DNA gyrase, topo IV, and in an in vitro transcription/translation assay.

Compd.	IC_50_ (μM)		
	DNA Gyrase	Topo IV	Inhib. of Protein Synthesis
OBP-4	1–5	10–15	5
OBP-5	20	>40	2
CIP	˂0.75	3–6	NT
LZD	NT	NT	5

NT, not tested.

## Supplementary Materials

^1^H-NMR,^13^C-NMR and HRMS spectra for oxazolidinone-fluoroquinolone hybrids OBP-(1–7) are available online.
