# Supplementary material for: Synthesis, Antibacterial Activities, Mode of Action and Acute Toxicity Studies of New Oxazolidinone-Fluoroquinolone Hybrids
Source: Molecules. 2019 Apr 25;24(8):1641. doi: 10.3390/molecules24081641 (PMC6514978; doi:10.3390/molecules24081641)
Supplement: Supplementary file 1 [file molecules-24-01641-s001.pdf]

Supplementary Material

Synthesis, Antibacterial Activities, Mode of Action and Acute Toxicity Studies of New Oxazolidinone-Fluoroquinolone Hybrids

Lili Liu [1,2,3], Liping Shao [1,2,3], Jing Li [4], Haifeng Cui [4], Bing Li [1,2,3], Xuzheng Zhou [1,2,3], Pengyue Lv [4] and Jiyu Zhang [1,2,3,*]

Figure S1. ^{1}H-NMR, ^{13}C-NMR and HRMS spectra of OBP-1

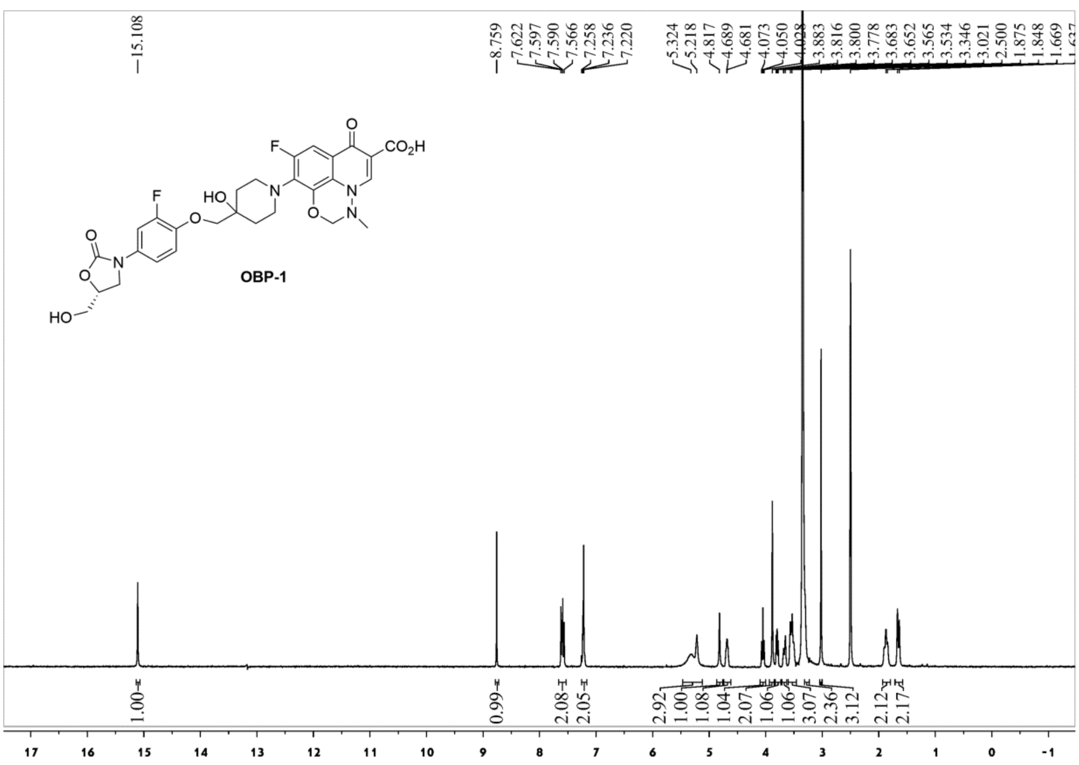

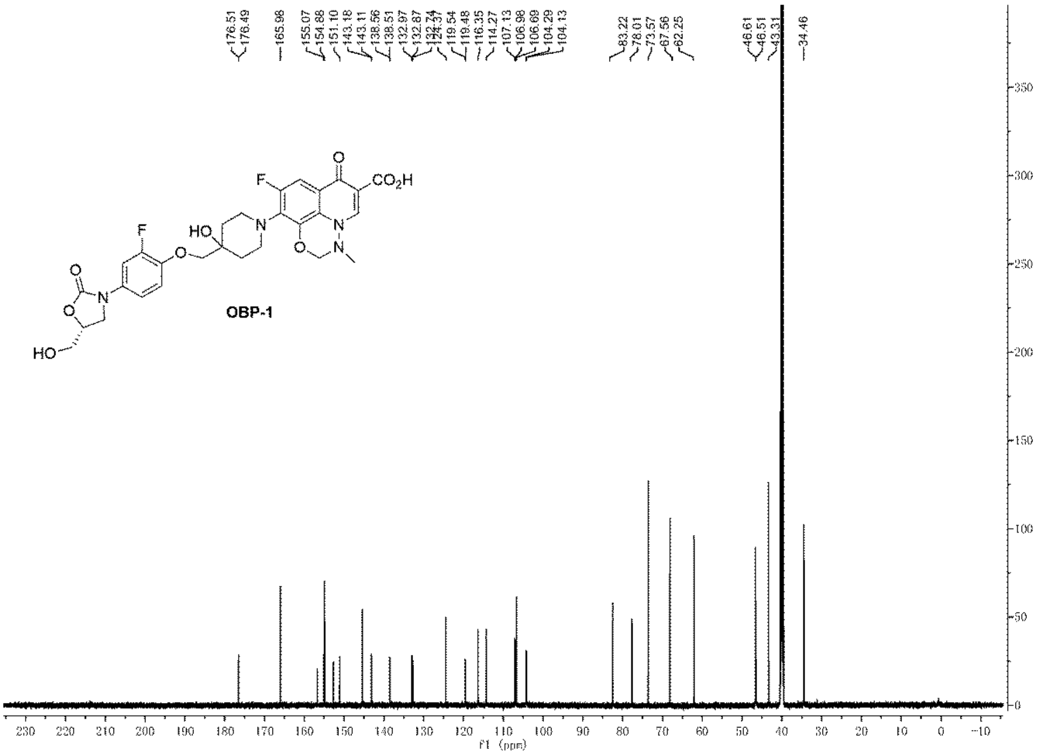
176.51
176.49
165.98
155.07
154.88
151.10
143.18
143.11
138.56
138.51
132.97
132.87
119.54
119.48
116.35
114.27
107.13
106.98
106.69
104.29
104.13
83.22
78.01
73.57
67.56
62.25
46.61
46.51
43.31
34.46
OBP-1
f1 (ppm)

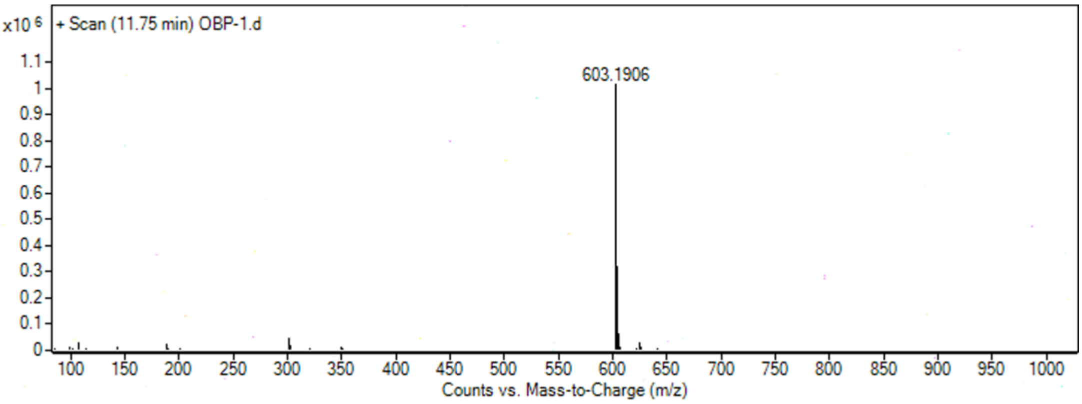
x10 6
+ Scan (11.75 min) OBP-1.d
603.1906
Counts vs. Mass-to-Charge (m/z)

Figure S2. ^{1}H-NMR, ^{13}C-NMR and HRMS spectra of OBP-2

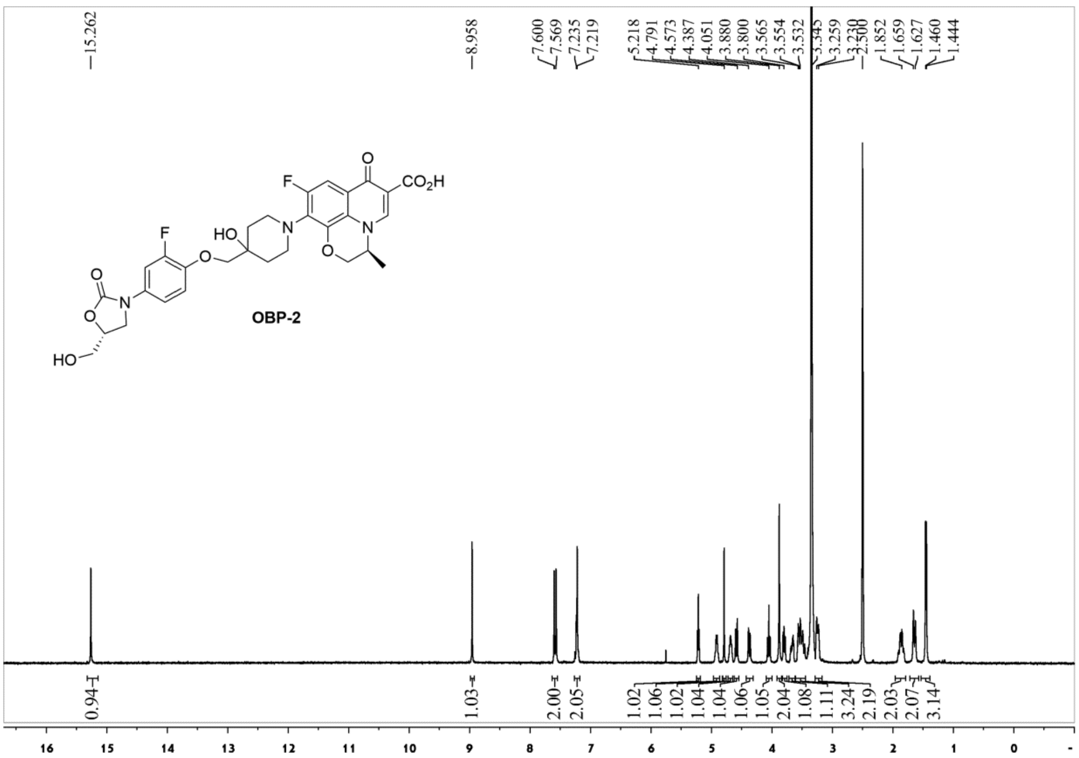

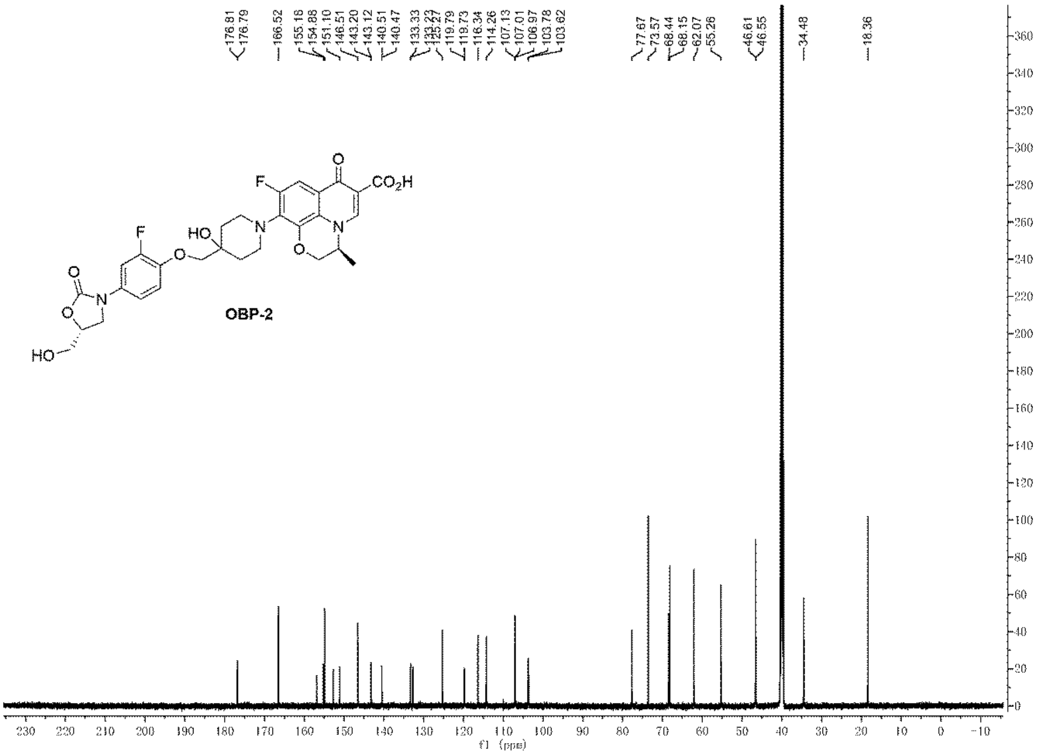

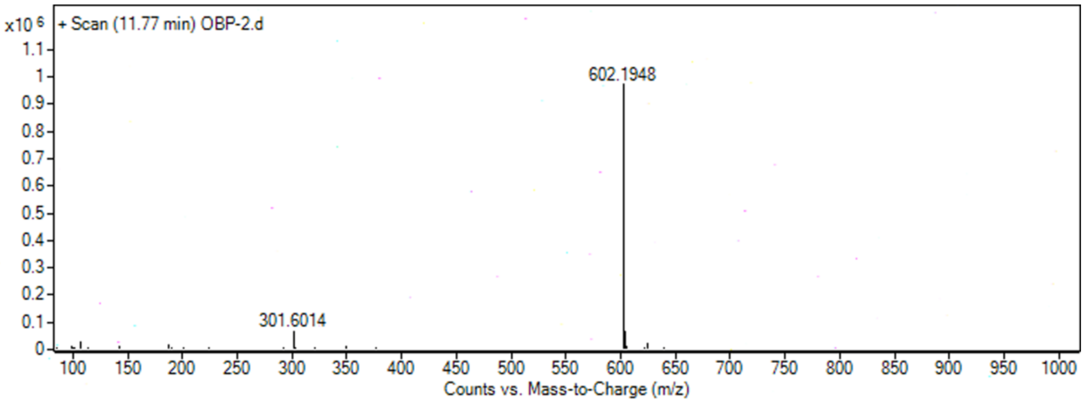
x10 6
+ Scan (11.77 min) OBP-2.d
602.1948
301.6014
1.1
1
0.9
0.8
0.7
0.6
0.5
0.4
0.3
0.2
0.1
0
100 150 200 250 300 350 400 450 500 550 600 650 700 750 800 850 900 950 1000
Counts vs. Mass-to-Charge (m/z)

Figure S3. **^{1}H-NMR, ^{13}C-NMR and HRMS spectra of OBP-3**

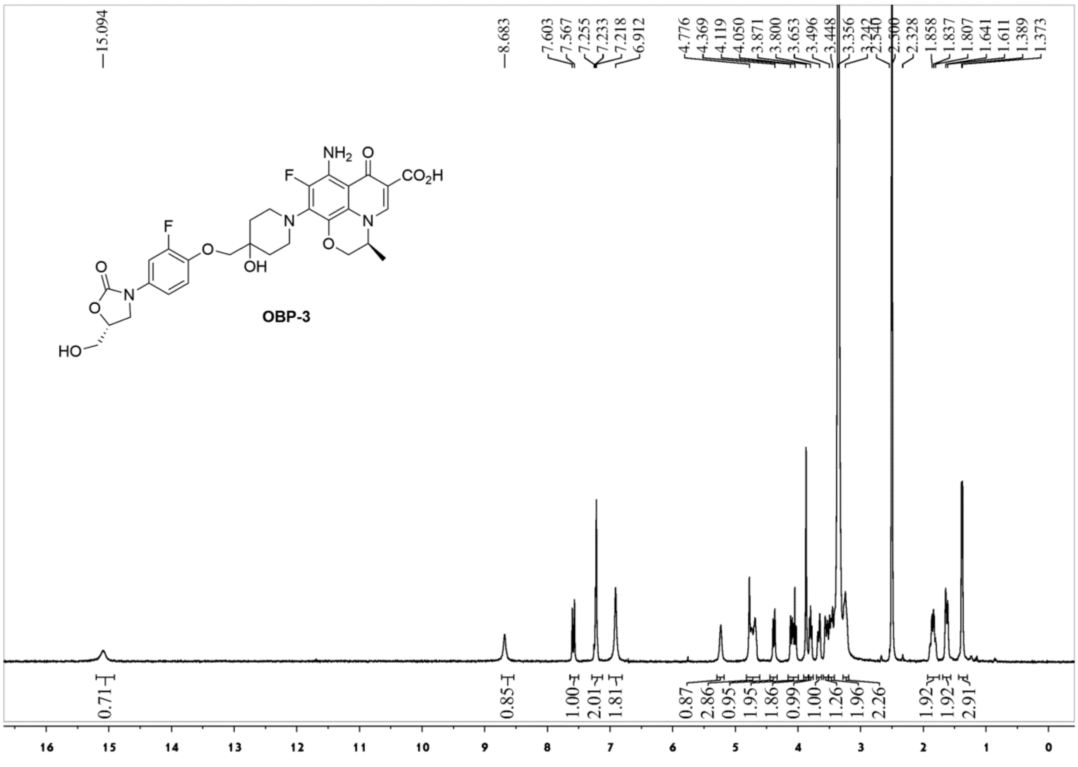

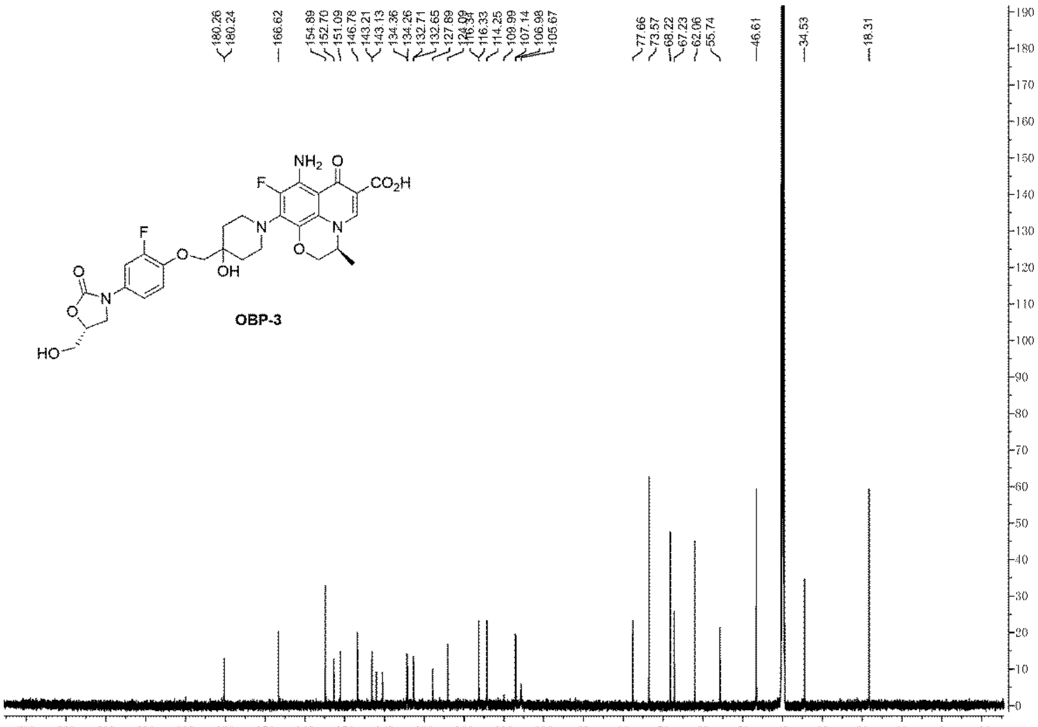

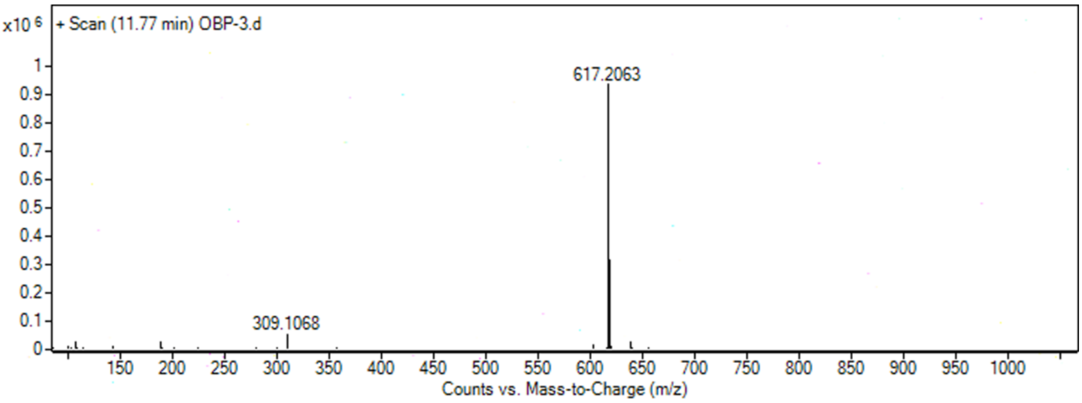

x10 6
+ Scan (11.77 min) OBP-3.d
617.2063
309.1068
1
0.9
0.8
0.7
0.6
0.5
0.4
0.3
0.2
0.1
0
150 200 250 300 350 400 450 500 550 600 650 700 750 800 850 900 950 1000
Counts vs. Mass-to-Charge (m/z)

Figure S4. ^{1}H-NMR, ^{13}C-NMR and HRMS spectra of OBP-4

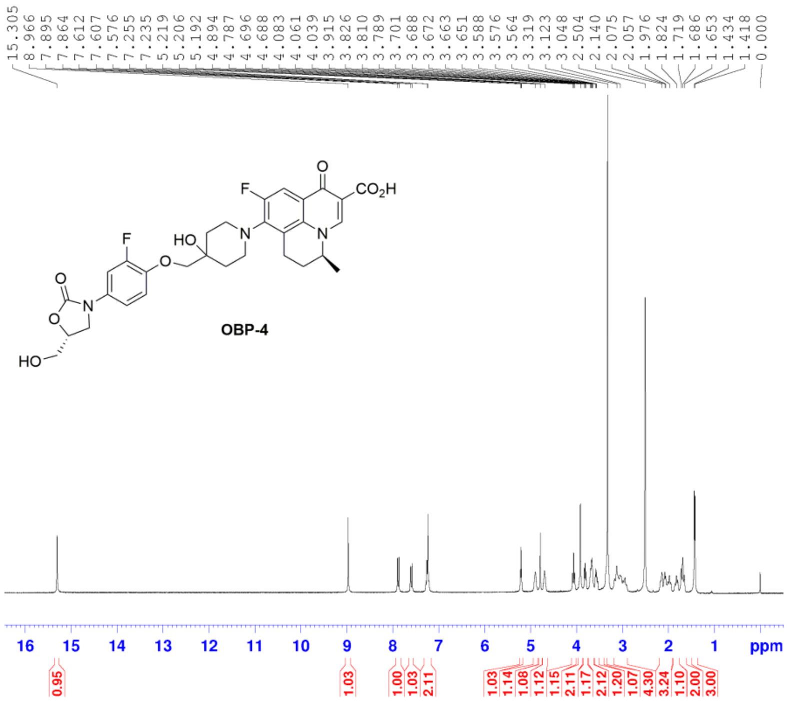

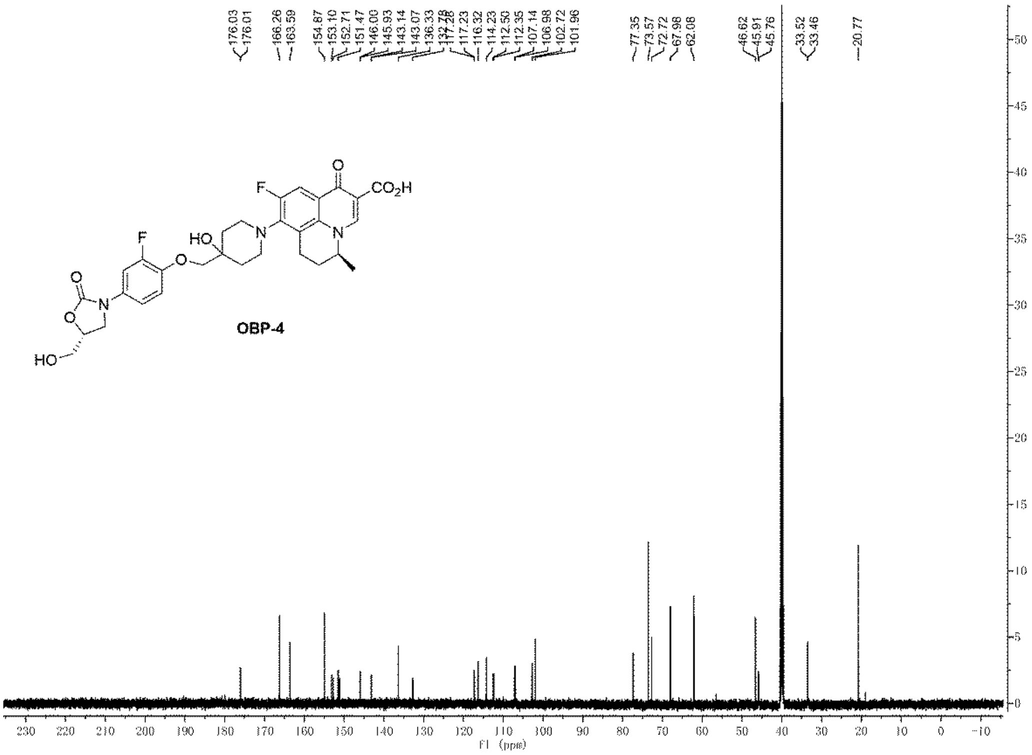

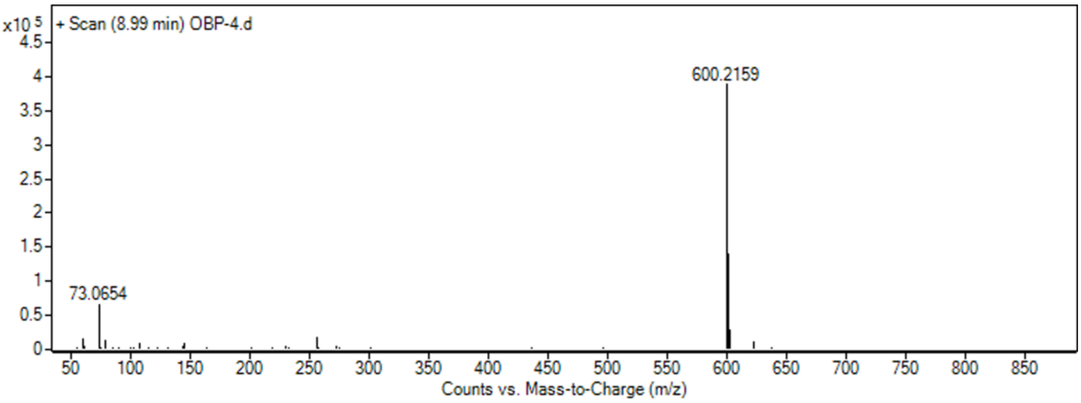
x10 5
+ Scan (8.99 min) OBP-4.d
600.2159
73.0654
Counts vs. Mass-to-Charge (m/z)

Figure S5. ^{1}H-NMR , ^{13}C-NMR and HRMS spectra of OBP-5

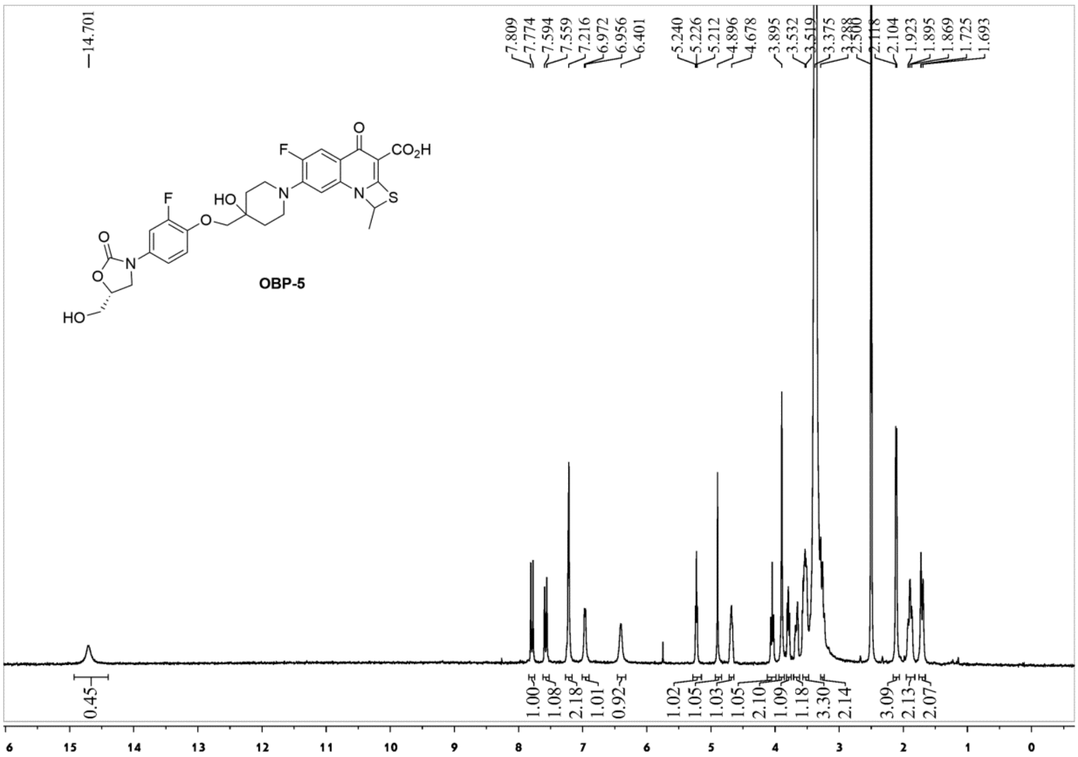

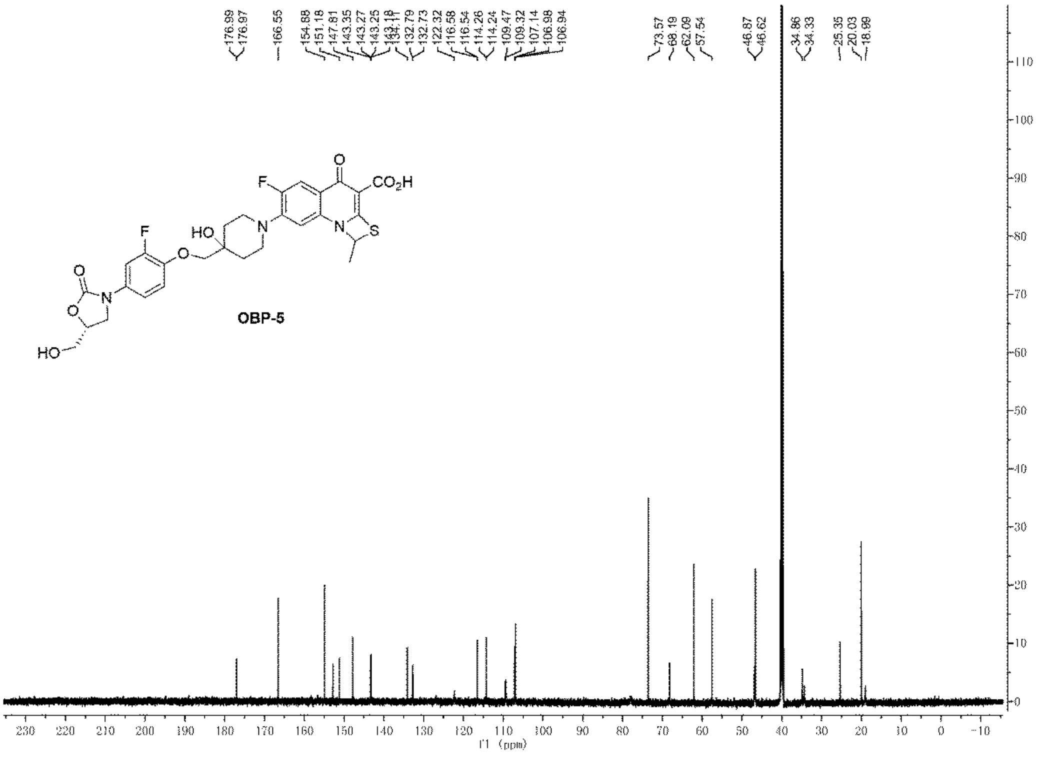

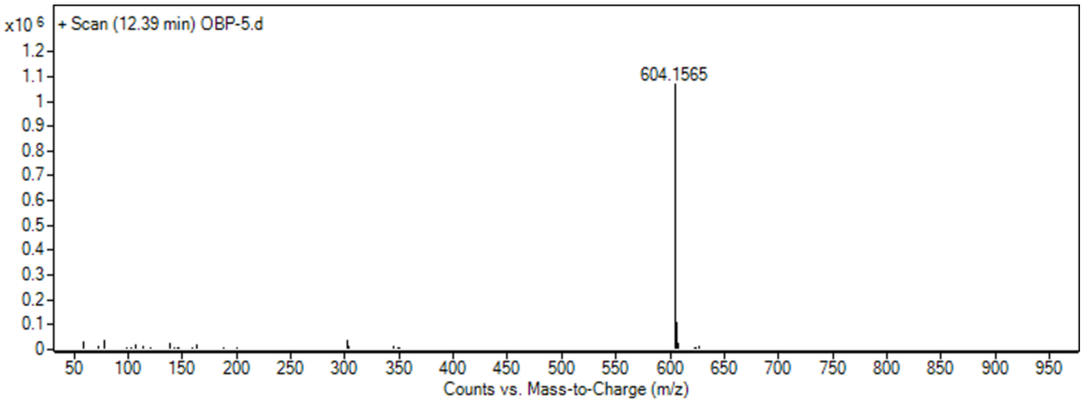
x10 6
+ Scan (12.39 min) OBP-5.d
604.1565
1.2
1.1
1
0.9
0.8
0.7
0.6
0.5
0.4
0.3
0.2
0.1
0
50 100 150 200 250 300 350 400 450 500 550 600 650 700 750 800 850 900 950
Counts vs. Mass-to-Charge (m/z)

Figure S6. **^{1}H-NMR, ^{13}C-NMR and HRMS spectra of OBP-6**

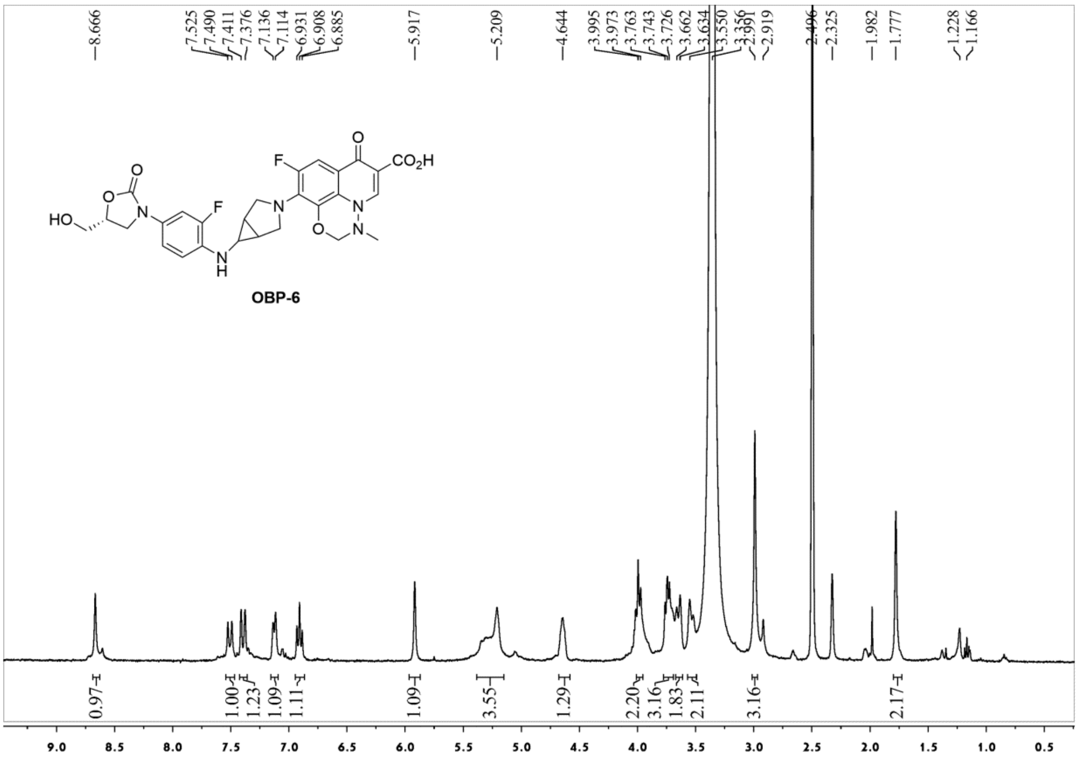

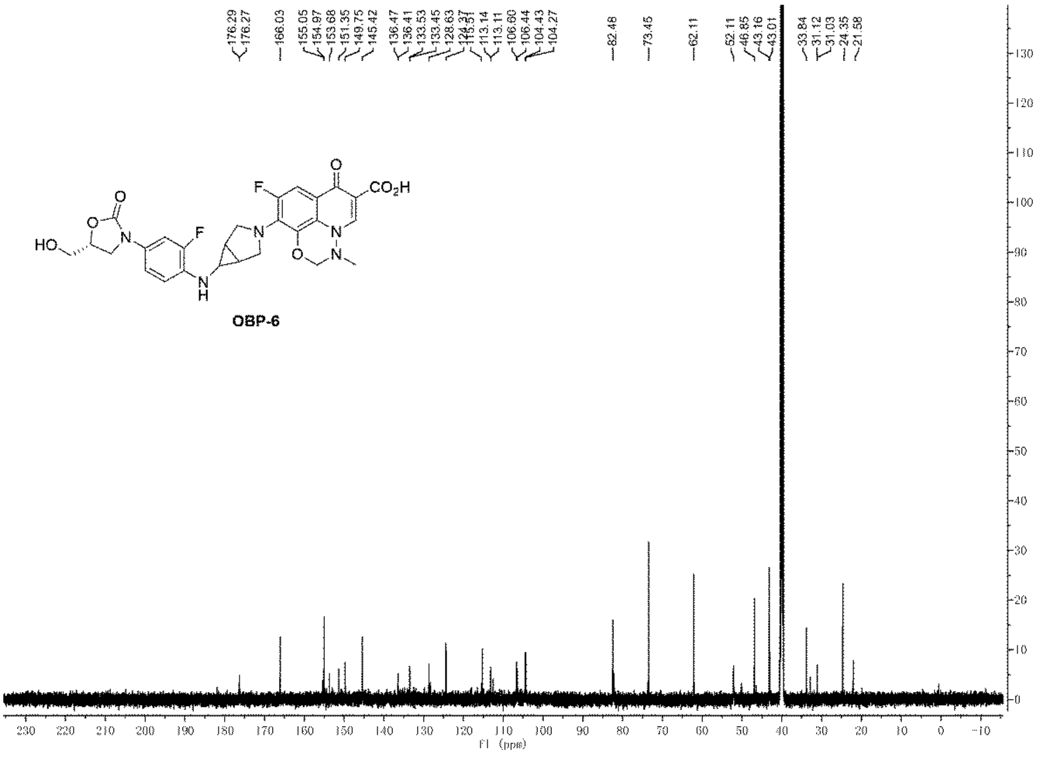

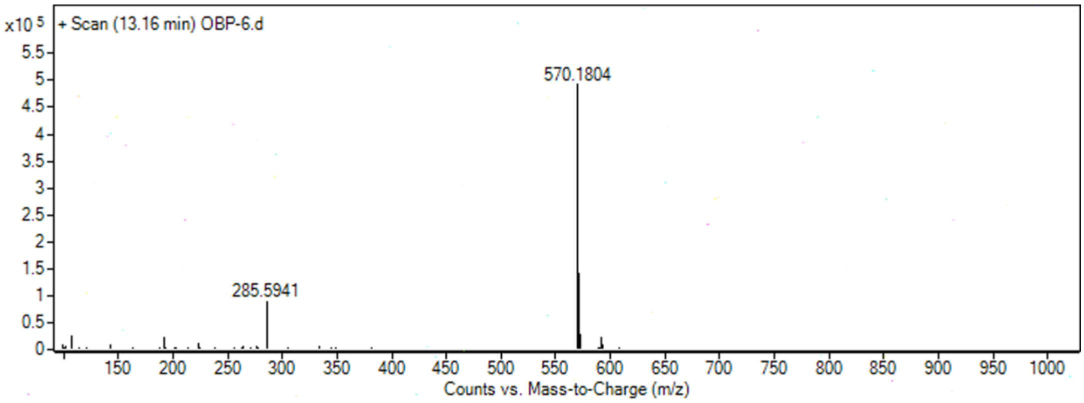
x10 5
+ Scan (13.16 min) OBP-6.d
570.1804
285.5941
Counts vs. Mass-to-Charge (m/z)

Figure S7. ^{1}H-NMR, ^{13}C-NMR and HRMS spectra of OBP-7

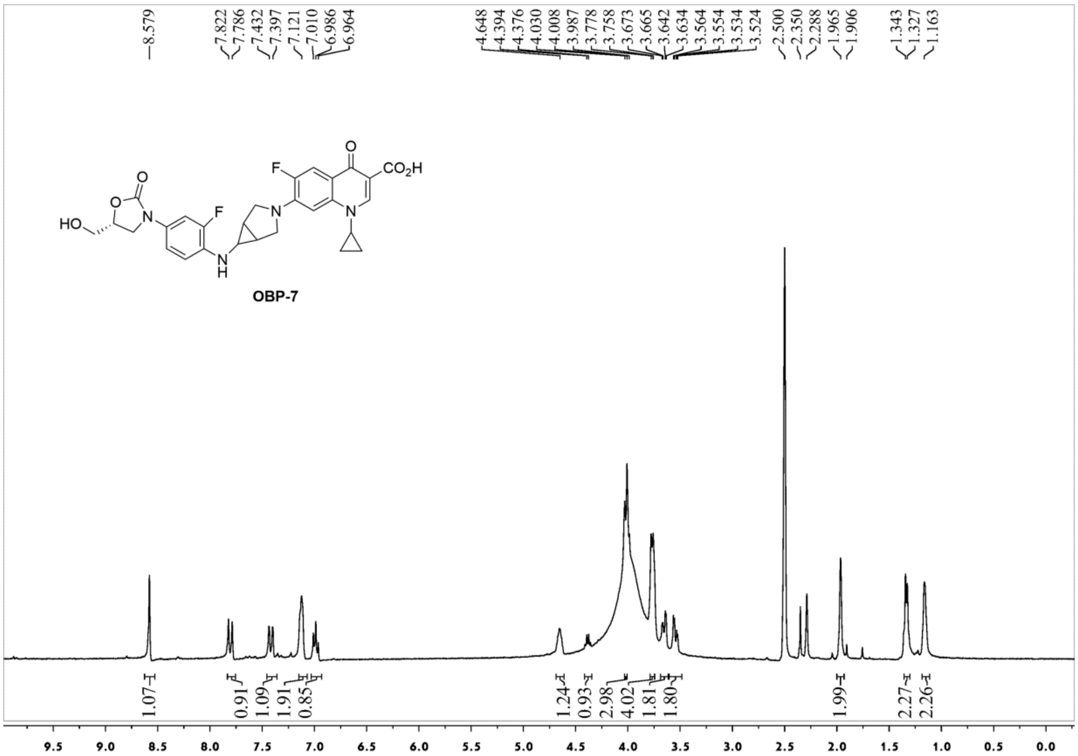

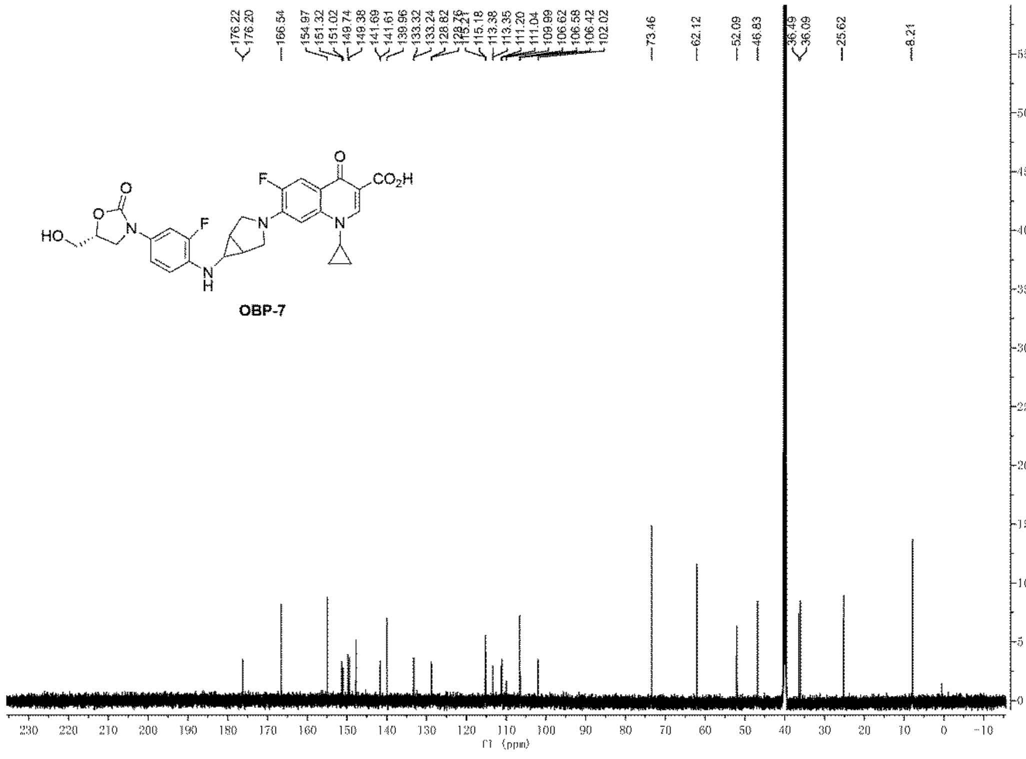

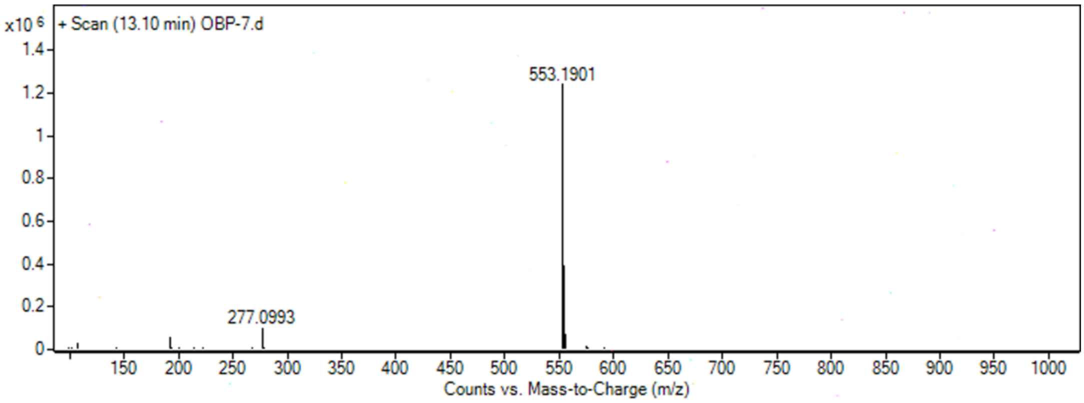
x10 6
+ Scan (13.10 min) OBP-7.d
553.1901
277.0993
1.4
1.2
1
0.8
0.6
0.4
0.2
0
150 200 250 300 350 400 450 500 550 600 650 700 750 800 850 900 950 1000
Counts vs. Mass-to-Charge (m/z)
